# Synthesis of Six-Membered *N*-Heterocyclic Carbene Precursors Based on Camphor

**DOI:** 10.3390/molecules28247973

**Published:** 2023-12-06

**Authors:** Jan Šegina, Luka Ciber, Helena Brodnik, Franc Požgan, Jurij Svete, Bogdan Štefane, Uroš Grošelj

**Affiliations:** Faculty of Chemistry and Chemical Technology, University of Ljubljana, Večna Pot 113, 1000 Ljubljana, Slovenia; js0639@student.uni-lj.si (J.Š.); luka.ciber@fkkt.uni-lj.si (L.C.); franc.pozgan@fkkt.uni-lj.si (F.P.); jurij.svete@fkkt.uni-lj.si (J.S.); bogdan.stefane@fkkt.uni-lj.si (B.Š.)

**Keywords:** ketopinic acid, amidation, reduction, diamines, *N*-heterocyclic carbene precursors, asymmetric catalysis, amidinium salts, hydrolysis

## Abstract

The *endo*- and *exo*-*N*-heterocyclic carbene precursors based on camphor were prepared diastereoselectively in five synthetic steps starting from (1*S*)-(+)-ketopinic acid. The obtained *N*-heterocyclic carbene precursors were investigated in an asymmetric benzoin reaction. All new compounds were fully characterized, and the absolute configurations were determined via X-ray diffraction and NOESY measurements.

## 1. Introduction

**Figure 1:**
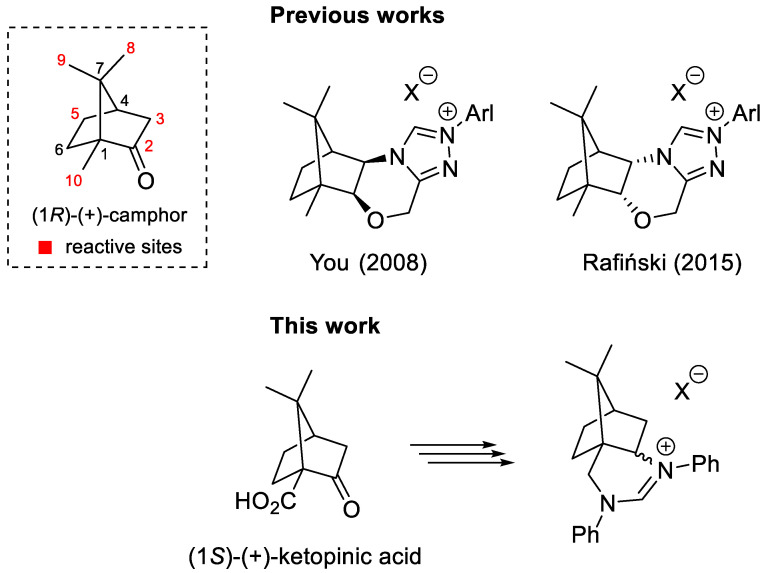
Efficient camphor-derived *N*-heterocyclic carbene precursors (**top**); six-membered *N*-heterocyclic carbene precursors (**bottom**) [13,19].

Within the chiral pool of building blocks, camphor is a privileged scaffold available in both enantiomeric forms. Camphor undergoes a wide range of different chemical transformations. These include rearrangements, such as the Wagner–Meerwein rearrangement and fragmentation reactions. In addition, these reactions enable the functionalization of apparently inactivated (remote) positions (Figure 1). Accordingly, they enable the synthesis of structurally and functionally very different products [1,2].

Numerous camphor derivatives have found their application in the field of asymmetric synthesis and catalysis. For example, camphorsultam has been widely used as an efficient chiral auxiliary [3,4], while the α-aminoisoborneol derivatives DAIB [5] and MIB [6] have been used as efficient ligands for the enantioselective addition of organozinc reagents to aldehydes. In the field of asymmetric organocatalysis [7,8], the first efficient camphor-derived organocatalyst was published in 2005 [9]. Both covalent and non-covalent organocatalysts based on camphor backbone were developed [10]. The first efficient *N*-heterocyclic carbene (NHC) organocatalyst was introduced by Enders and Kallfass in 2002 [11], and efficient camphor-based NHC analogs appeared in 2008 [12,13,14,15,16,17,18,19,20]. The NHC precursors developed by You [13] and Rafiński [19] are particularly efficient in enantioselective catalysis (Figure 1).

Recently, we prepared camphor-derived 1,2-, 1,3-, and 1,4-diamines as potential building blocks for bifunctional organocatalysts with camphor as the exclusive chiral scaffold [21]. These camphor-derived diamines were used for the preparation of bifunctional non-covalent thiourea and squaramide organocatalysts [21,22] and bifunctional quaternary ammonium salt phase transfer organocatalysts [23]. The camphor-1,3-diamine-derived squaramide organocatalyst exhibited excellent catalytic activity in enantioselective conjugative additions of 1,3-dicarbonyls and α-amino acid-derived pyrrolin-4-ones to *trans*-β-nitrostyrenes [22,24]. In extension, we reasoned that camphor-based 1,3-diamines [21,22] could be transformed into cyclic amidinium salts as interesting non-racemic precursors of NHC. In this article, we report the synthesis of a novel type of six-membered NHC precursors in five steps from commercially available (1*S*)-(+)-ketopinic acid (Figure 1).

## 2. Results and Discussion

### 2.1. Synthesis

**Scheme 1:**
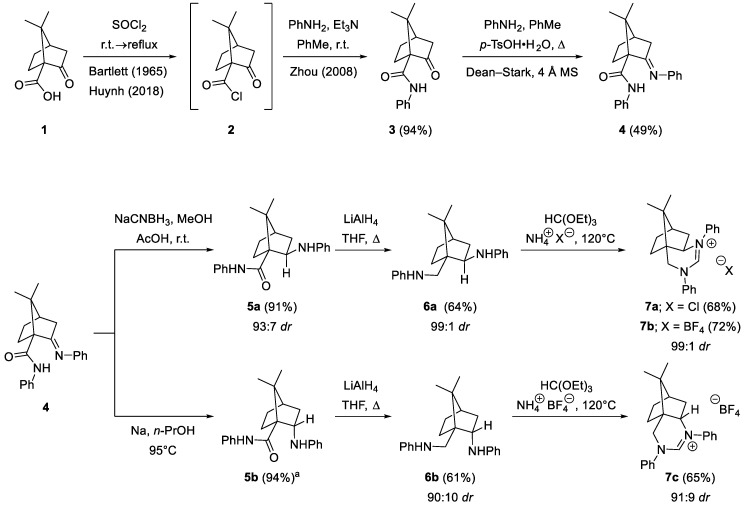
Synthesis of NHC precursors **7a**–**c**; ^a^ *dr* could not be determined [25,26,27,28].

The starting point for the synthesis was commercially available (1*S*)-(+)-ketopinic acid (**1**) (Scheme 1), which can alternatively be prepared from the much cheaper (1*S*)-(+)-10-camphorsulfonic acid according to procedures described in the literature [25,26,27]. First, (1*S*)-(+)-ketopinic acid (**1**) was treated with thionyl chloride. After the removal of volatiles, crude acid chloride **2** was reacted with aniline in the presence of excess triethylamine in anhydrous toluene to give amide **3** [28] in 94% yield. The treatment of ketone **3** with excess aniline in the presence of catalytic amounts of *para*-toluenesulfonic acid with azeotropic removal of water using 4 Å molecular sieves gave imine **4** in 49% yield. Attempts to reduce both amide and imine functionality in one step to obtain diamines **6a**/**6b** with excess LiAlH_4_ or BH_3_•THF resulted in complex product mixtures. Therefore, sequential reduction was performed. A diastereoselective reduction in imine **4** with NaCNBH_3_ [29] in methanol in the presence of acetic acid afforded *exo*-aminoamide **5a** a 91% yield with high diastereoselectivity (*dr* 93:7). A reduction in imine **4** with sodium [21,22] in *n*-propanol at 95 °C gave a mixture of products containing *endo*-epimer **5b** in an estimated 94% combined yield. The diastereoselectivity of this reduction could not be determined. A reduction in imine **4** with Zn in the presence of KOH and catalytic hydrogenation with Pd-C in methanol failed. A reduction in epimeric amides **5a** and **5b** with excess LiAlH_4_ gave diamines **6a** and **6b** in 64% and 61% yields, respectively. Diamines **6a** and **6b** were isolated with a diastereoselectivity of 99:1 and 90:10, respectively. Finally, cyclic amidinium salts **7a**–**c** were isolated in 65–72% yield by treating diamines **6a** and **6b** in triethyl orthoformate in the presence of ammonium tetrafluoroborate or ammonium chloride at an elevated temperature [30,31]. The *exo*-amidinium salts **7a** and **7b** were isolated in 99:1 *dr*, while the *endo*-salt **7c** was isolated in 91:9 *dr* (Scheme 1). The recrystallization of **7c** from *i*-PrOH did not improve the diastereomeric ratio.

### 2.2. Structure Determination

The structures of novel camphor derivatives **4**–**8** were determined using spectroscopic methods (^1^H and ^13^C NMR, 2D NMR, HRMS, and IR). The *endo*-amine **5b** could not be isolated in pure form. It was used directly in further transformation.

**Figure 2:**
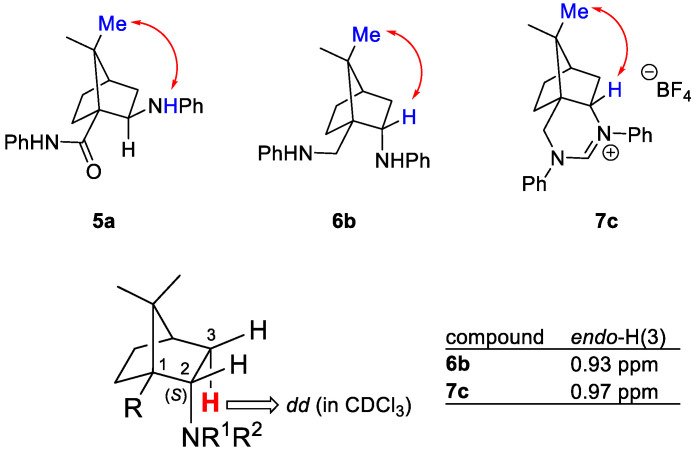
Determination of the absolute configuration at the C-2 based on the observed NOE correlation spectroscopy cross peaks (**top**) and chemical shift correlations in the series of C-2 *endo*-isomers (**bottom**).

The configurations at the newly formed stereogenic centers (C-2) in *endo*-diamine **6b** and *endo*-amidine **7c** were determined using NOESY spectroscopy. The cross-peak between the *exo*-H(2) and the Me-C(8) group was consistent with the (2*S*)-configuration (Figure 2). Similarly, the (2*R*)-configuration at C-2 in *exo*-camphoramine **5a** was in line with the NOE between the Me-C(8) and the *exo*-NH group (see Supplementary Materials). The *endo*-stereochemistry of diamine **6b** and cyclic amidine **7c** was additionally confirmed on the basis of a chemical shift and multiplicity of the *endo*-H-C(3), which appear as a doublet of doublet at 0.93 ppm (**6b**) and 0.97 ppm (**7c**) (Figure 2) ([21,23], see Supplementary Materials).

**Figure 3:**
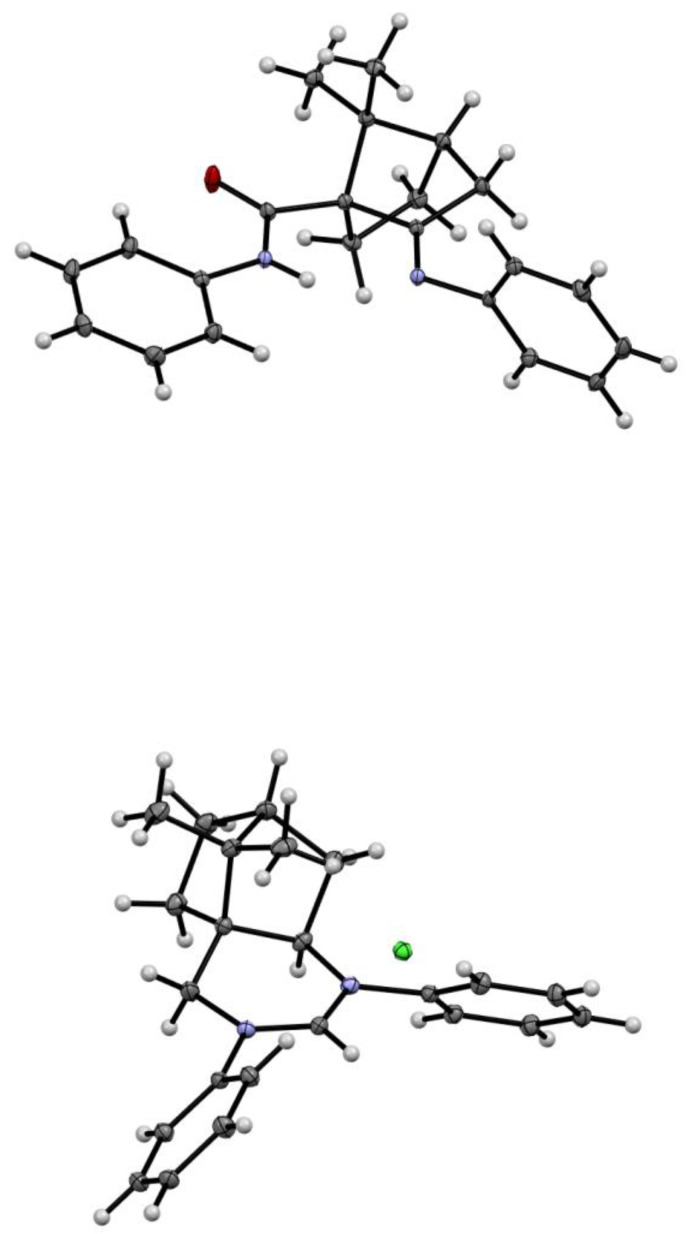
Molecular structures of compounds **4** (**top**) and **7a** (**bottom**). Thermal ellipsoids are shown at a 50% probability.

Finally, the structures of compounds **4** and **7a** were unambiguously determined using X-ray diffraction (Figure 3) (see Supplementary Materials).

### 2.3. Performance of Camphor-Derived NHC Precursors in Benzoin Reaction

**Scheme 2:**
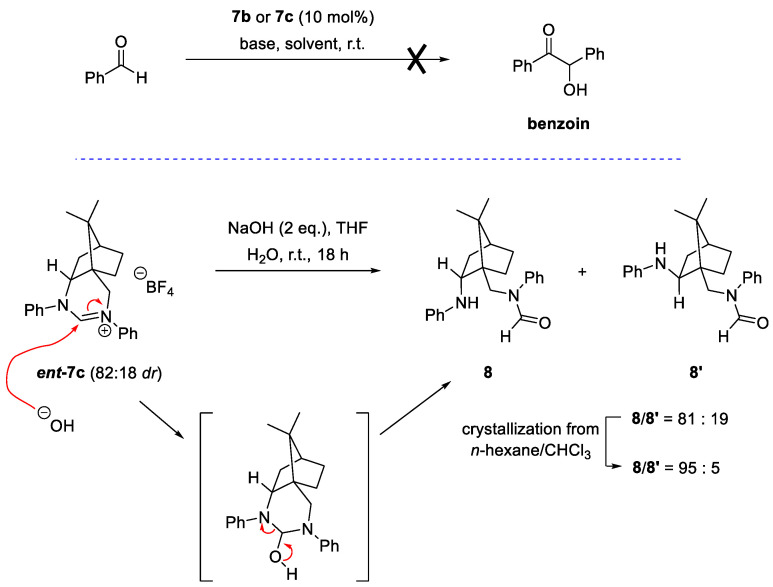
Benzoin reaction (**top**) and opening of amidinium salt **7c** (**bottom**).

**Figure 4:**
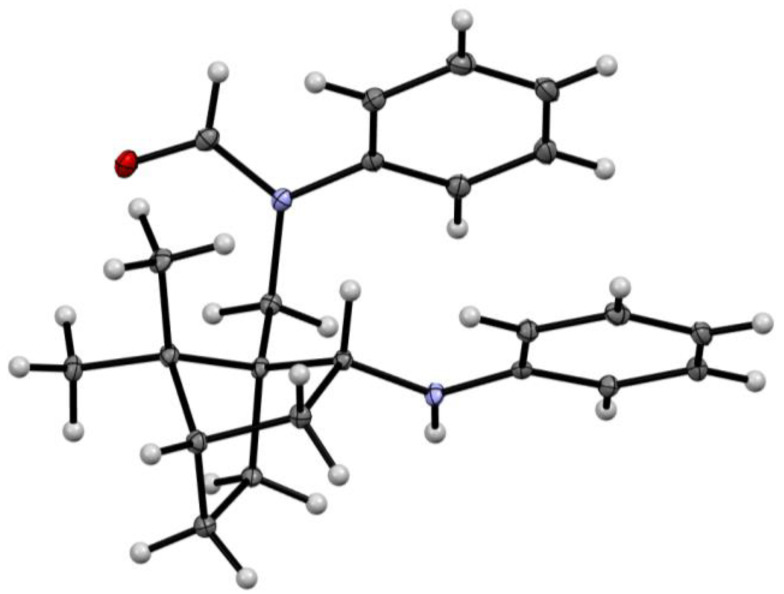
The molecular structure of compound **8**. Thermal ellipsoids are shown at a 50% probability.

The model reaction for evaluating the efficiency of the procatalyst **7a**–**c** was the benzoin reaction with benzaldehyde, in which 10 mol% of the procatalyst was used (Scheme 2). Various bases were used for the in situ formation of the nucleophilic carbene catalyst. With aqueous Na_2_CO_3_ [32] as a base, no reaction took place, and the NHC precursors remained unchanged. Since the estimated pKa values of amidinium salts **7a**–**c** were in the range of 24 to 26 [33,34,35], stronger bases were used to obtain the NHCs. Reactions in the presence of *t*BuONa, LiHDMS, and LDA in anhydrous THF or 1,4-dioxane [36] did not give the benzoin product, while the amidinium salts **7a**–**c** decomposed, presumably due to the traces of water present in the reaction mixture or during workup. The attempts to confirm the formation of the carbene catalyst in situ (in the NMR tube) were also unsuccessful, and only the decomposition products were observed. To verify the hydrolysis of amidinium salt **7** under basic conditions and to identify the decomposition product, the amidinium salt ***ent*-7c** (*dr* = 82:18; prepared from (1*R*)-(−)-ketopinic acid) was hydrolyzed in a mixture of THF and water with two equivalents of NaOH. After 18 h, the amidinium starting salt ***ent*-7c** was quantitatively hydrolyzed to an aminoamide mixture **8**/**8**′ in the ratio 81:19. The crystallization of the crude product yielded single crystals of **8** (*dr* = 95:5) suitable for X-ray analysis, which confirmed the structure of hydrolyzed product **8** (Figure 4). The absolute configuration at position 2 was additionally confirmed using the NOESY measurement ([21,23], see Supplementary Materials) Amidinium salts ***ent*-7c** are hydrolytically unstable even in the presence of traces of water and are, therefore, not suitable as precursors of NHCs under applied reaction conditions.

## 3. Materials and Methods

### 3.1. Materials and General Methods

The solvents for the extractions and chromatography were of technical quality and distilled prior to use. The extracts were dried over technical-grade anhydrous Na_2_SO_4_. Melting points were determined on a Kofler micro hot stage and using the SRS OptiMelt MPA100—Automated Melting Point System (Stanford Research Systems, Sunnyvale, CA, USA). IR spectra were recorded using a Perkin-Elmer Spectrum BX FTIR spectrophotometer (PerkinElmer, Waltham, MA, USA). Mass spectra were recorded using an Agilent 6224 Accurate Mass TOF LC/MS (Agilent Technologies, Santa Clara, CA, USA). NMR spectra were recorded with a Bruker UltraShield 500 plus (Bruker, Billerica, MA, United States) at 500 MHz for the ^1^H nucleus and 126 MHz for the ^13^C nucleus using CDCl_3_ with TMS as the internal standard solvent. Column chromatography (CC) was performed on silica gel (Silica gel 60, particle size: 0.035–0.070 mm (Sigma-Aldrich, St. Louis, MO, USA)). All commercially available chemicals used were purchased from Sigma-Aldrich (St. Louis, MO, USA).

#### 3.1.1. Synthesis of (1*R*,4*R*)-7,7-Dimethyl-2-oxo-*N*-phenylbicyclo[2.2.1]heptane-1-carboxamide (**3**) [28]

SOCl_2_ (8 mL) was added to the flask containing (1*S*)-(+)-ketopinic acid (**1**) (20 mmol, 3.644 g) under argon. The reaction mixture was stirred for 2 h at room temperature and then for 1 h under reflux. Excess SOCl_2_ was evaporated in vacuo. The crude acid chloride **2** was immediately reacted further.

A solution of acid chloride **2** (20 mmol) in anhydrous toluene at 0 °C (20 mL), dropwise aniline (20 mmol, 1.823 mL) and Et_3_N (7 mL) were added. The reaction mixture was stirred at room temperature for 20 h. Ethyl acetate (20 mL) was added to the reaction mixture, followed by extraction with NaCl (aq. sat., 2 × 10 mL). The organic phase was dried over anhydrous Na_2_SO_4_, filtered, and the volatiles were evaporated in vacuo. The crude amide **3** was further reacted without additional purification. The crude amide **3** can, if needed, be purified via recrystallization from EtOH. Yield: 3.838 g (18.8 mmol, 94%) of white solid. ^1^H-NMR (500 MHz, CDCl_3_) *δ* (ppm): 1.04 (*s*, 3H) 1.34 (*s*, 3H), 1.45–1.52 (*m*, 1H), 1.68–1.75 (*m*, 1H), 2.05 (*d*, *J* = 18.8 Hz, 1H), 2.12 (*t*, *J* = 4.5 Hz, 1H), 2.16–2.25 (*m*, 1H), 2.55–2.65 (*m*, 2H), 7.07–7.12 (m, 1H), 7.29–7.35 (*m*, 2H), 7.59–7.64 (*m*, 2H), 9.70 (br *s*, 1H).

#### 3.1.2. Synthesis of (1*R*,4*R*,*E*)-7,7-Dimethyl-*N*-phenyl-2-(phenylimino)bicyclo[2.2.1]heptane-1-carboxamide (**4**)

To a solution of amide **3,** (10 mmol, 2.573 g) in anhydrous toluene (40 mL) under argon were added aniline (50 mmol, 4.556 mL) and *para*-toluenesulfonic acid monohydrate (2 mmol, 380 mg). The flask was fitted with a Dean–Stark trap filled with activated 4 Å molecular sieves and a reflux condenser. The reaction mixture was refluxed for 20 h. In a cooled reaction mixture, EtOAc (30 mL) and H_2_O (30 mL) were added, and the phases were separated. The aqueous phase was extracted with EtOAc (2 × 20 mL). The combined organic phase was washed with brine (10 mL), dried under anhydrous Na_2_SO_4_, filtered, and the volatiles were evaporated in vacuo. The crude product was purified using CC (Silica gel 60, EtOAc/petroleum ether = 1:5). The fractions containing pure product **4** were combined, and the volatiles were evaporated in vacuo. Yield: 1.629 g (4.9 mmol, 49%) of orange solid; mp = 138–140 °C. [*α*]_D_^r.t.^ = +76.9 (0.13, CHCl_3_). EI-HRMS: *m*/*z* = 333.1956 (MH^+^); C_22_H_25_N_2_O requires: *m*/*z* = 333.1961 (MH^+^); *ν*_max_ 3238, 3177, 3116, 3022, 3000, 2970, 2954, 1680, 1594, 1547, 1487, 1455, 1443, 1391, 1375, 1322, 1297, 1253, 1229, 1206, 1196, 1154, 1102, 1073, 1041, 1026, 966, 905, 885, 870, 833, 816, 790, 760, 712, 695, 662 cm^−1^. ^1^H-NMR (500 MHz, CDCl_3_) *δ* (ppm): 1.05 (*s*, 3H), 1.37 (*s*, 3H), 1.34–1.41 (*m*, 1H), 1.83 (*ddd*, *J* = 4.8, 9.4, 13.7 Hz, 1H), 1.91–1.99 (*m*, 2H), 2.10–2.19 (*m*, 1H), 2.41 (*dt*, *J* = 4.1, 18.1, 1H), 2.67–2.76 (*m*, 1H), 6.81–6.91 (*m*, 2H), 7.03–7.09 (*m*, 1H), 7.13–7.19 (*m*, 1H), 7.26–7.34 (*m*, 2H), 7.35–7.42 (*m*, 2H), 7.58–7.67 (*m*, 2H), 11.49 (*s*, 1H). ^13^C-NMR (126 MHz, CDCl_3_) *δ* (ppm): 19.64, 20.51, 27.58, 30.56, 35.99, 43.28, 50.25, 60.70, 118.93, 119.26, 119.35, 123.05, 123.82, 128.10, 128.13, 128.64, 128.67, 128.70, 137.88, 148.56, 168.45, 182.04.

#### 3.1.3. Synthesis of (1*R*,2*R*,4*R*)-7,7-Dimethyl-*N*-phenyl-2-(phenylamino)bicyclo[2.2.1]heptane-1-carboxamide (**5a**)

NaCNBH_3_ (12 mmol, 794 mg, ω = 0.95) was added to a solution of imine **4** (332 mg, 1 mmol) in anhydrous MeOH (15 mL) under argon. Then, a catalytic amount of anhydrous acetic acid (0.2 mL) was added, and the reaction mixture was stirred at room temperature for 5 h. The reaction was stopped by adding a saturated solution of NaHCO_3_ (5 mL) and EtOAc (10 mL), and the phases were separated. The aqueous phase was extracted with EtOAc (10 mL), and the combined organic phase was washed with brine (5 mL), dried over anhydrous Na_2_SO_4_, which was filtered, and the volatiles were evaporated in vacuo. Yield: 304 mg (0.91 mmol, 91%, *dr* 93:7) of a dirty white solid; mp = 174–175 °C. [*α*]_D_^r.t.^ = −10.6 (0.12, CHCl_3_). EI-HRMS: *m*/*z* = 335.2112 (MH^+^); C_22_H_27_N_2_O requires: *m*/*z* = 335.2118 (MH^+^); *ν*_max_ 3333, 2954, 1652, 1601, 1519, 1498, 1439, 1388, 1308, 1245, 1180, 1104, 1072, 869, 746, 690 cm^−1^. ^1^H-NMR (500 MHz, CDCl_3_) *δ* (ppm): 1.10 (*s*, 3H), 1.22–1.29 (*m*, 1H), 1.31 (*s*, 3H), 1.46–1.53 (*m*, 1H), 1.81–1.87 (*m*, 1H), 1.89 (*t*, *J* = 4.3 Hz, 1H), 1.91–1.98 (*m*, 1H), 2.15 (*dd*, *J* = 8.6, 12.8 Hz, 1H), 2.58 (*td*, *J* = 4.6, 12.4 Hz, 1H), 3.58 (*dt*, *J* = 5.0, 9.3 Hz, 1H), 4.09 (*d*, *J* = 4.9 Hz, 1H), 6.62–6.68 (*m*, 2H), 6.74–6.79 (*m*, 1H), 6.97–7.02 (*m*, 1H), 7.13–7.23 (*m*, 6H), 8.92 (br *s*, 1H). ^13^C-NMR (126 MHz, CDCl_3_) *δ* (ppm): 21.48, 21.50, 26.91, 31.65, 42.35, 45.79, 50.79, 58.43, 63.09, 114.36, 119.09, 120.52, 123.98, 128.71, 129.52, 138.04, 147.47, 171.17.

#### 3.1.4. Synthesis of (1*R*,2*S*,4*R*)-7,7-dimethyl-*N*-phenyl-2-(phenylamino)bicyclo[2.2.1]heptane-1-carboxamide (**5b**)

Imine **4** (3 mmol, 997 mg) was dissolved in *n*-PrOH (100 mL), and the mixture was heated to 95 °C. Then, the first sodium piece was added to the reaction mixture, followed by another sodium piece after the first sodium piece had reacted, then the third, and so on. After 2 h at 95 °C, when the last sodium piece had reacted, H_2_O (100 mL) and Et_2_O (100 mL) were added to the cooled reaction mixture, and the phases were separated. The aqueous phase was extracted with Et_2_O (2 × 100 mL), and the combined organic phase was dried over anhydrous Na_2_SO_4_, filtered, and the volatiles were evaporated in vacuo. The crude amine **5b** was further reacted without additional purification. Yield: 943 mg (2.82 mmol, 94%) of grey oil.

#### 3.1.5. Synthesis of (1*S*,2*R*,4*R*)-7,7-Dimethyl-*N*-phenyl-1-((phenylamino)methyl)bicyclo[2.2.1]-heptan-2-amine (**6a**)

To a solution of compound **5a** (0.6 mmol, 201 mg) in anhydrous THF (2 mL) under argon at room temperature was added LiAlH_4_ (2.4 M in THF, 1.0 mL) dropwise. After this addition, the reaction mixture was stirred for 20 h at 60 °C. The reaction was cooled (0 °C) and quenched by the careful addition of a mixture of H_2_O and THF in a 1:5 ratio. The reaction mixture was filtered, and the cake was washed with EtOAc (3 × 15 mL). The collected liquid was dried over anhydrous Na_2_SO_4_, filtered, and the volatiles were evaporated in vacuo. The crude product was purified using CC (Silica gel 60, EtOAc/petroleum ether = 1:10). The fractions containing the pure product **6a** were combined and the volatiles were evaporated in vacuo. Yield: 123 mg (0.384 mmol, 64%, *dr* 99:1) of dirty white semisolid. [*α*]_D_^r.t.^ = −123.7 (0.12, CHCl_3_). EI-HRMS: *m*/*z* = 321.2323 (MH^+^); C_22_H_29_N_2_ requires: *m*/*z* = 321.2325 (MH^+^); *ν*_max_ 3412, 3050, 2951, 2876, 1600, 1499, 1429, 1387, 1369, 1302, 1251, 1180, 1152, 1096, 1073, 1028, 992, 866, 744, 689 cm^−1^. ^1^H-NMR (500 MHz, CDCl_3_) *δ* (ppm): 0.99 (*s*, 3H), 1.17 (*s*, 3H), 1.21–1.28 (*m*, 1H), 1.46–1.53 (*m*, 1H), 1.70–1.87 (*m*, 4H), 1.91–1.99 (*m*, 1H), 3.20 (*d*, *J* = 12.4 Hz, 1H), 3.29 (*d*, *J* = 12.3 Hz, 1H), 3.50–3.56 (*m*, 1H), 3.69 (br *s*, 1H), 4.05 (br *s*, 1H), 6.55–6.62 (*m*, 4H), 6.68 (*t*, *J* = 7.3 Hz, 2H), 7.08–7.19 (*m*, 4H). ^13^C-NMR (126 MHz, CDCl_3_) *δ* (ppm): 21.12, 21.24, 27.41, 34.22, 40.72, 43.58, 46.13, 47.69, 51.93, 59.63, 113.42, 113.53, 117.47, 117.64, 129.27, 129.39, 147.58, 149.05.

#### 3.1.6. Synthesis of (1*S*,2*S*,4*R*)-7,7-Dimethyl-*N*-phenyl-1-((phenylamino)methyl)bicyclo[2.2.1]-heptan-2-amine (**6b**)

To a solution of compound **5b** (0.5 mmol, 201 mg) in anhydrous THF (2 mL) under argon at room temperature was added LiAlH_4_ (2.4 M in THF, 1.0 mL) dropwise. After this addition, the reaction mixture was stirred for 20 h at 60 °C. The reaction was cooled (0 °C) and quenched by the careful addition of a mixture of H_2_O and THF in a 1:5 ratio. The reaction mixture was filtered, and the cake was washed with EtOAc (3 × 15 mL). The collected liquid was dried over anhydrous Na_2_SO_4_, filtered, and the volatiles were evaporated in vacuo. The crude product was purified using CC (Silica gel 60, EtOAc/petroleum ether = 1:10). The fractions containing pure product **6b** were combined, and the volatiles were evaporated in vacuo. Yield: 98 mg (0.305 mmol, 61%, *dr* 90:10) of grey semisolid. [*α*]_D_^r.t.^ = +25.0 (0.15, CHCl_3_). EI-HRMS: *m*/*z* = 321.2323 (MH^+^); C_22_H_29_N_2_ requires: *m*/*z* = 321.2325 (MH^+^); *ν*_max_ 3403, 3050, 3020, 2951, 2875, 1600, 1499, 1430, 1388, 1372, 1310, 1276, 1252, 1179, 1153, 1100, 1072, 1045, 1028, 992, 866, 744, 689, 617 cm^−1^. ^1^H-NMR (500 MHz, CDCl_3_) *δ* (ppm): 0.93 (*dd*, *J* = 3.8, 13.1 Hz, 1H), 1.03 (*s*, 3H), 1.08 (*s*, 3H), 1.29–1.36 (*m*, 1H), 1.61–1.69 (*m*, 1H), 1.73 (*t*, *J* = 4.6 Hz, 1H), 1.84–1.92 (m, 1H), 2.00–2.07 (*m*, 1H), 2.44–2.52 (*m*, 1H), 3.18 (*d*, *J* = 11.7 Hz, 1H), 3.24 (*d*, *J* = 11.7 Hz, 1H), 3.88–3.94 (*m*, 1H), 4.01 (*s*, 1H), 4.37 (*s*, 1H), 6.48–6.54 (*m*, 2H), 6.62–6.68 (*m*, 3H), 6.70–6.75 (*m*, 1H), 7.08–7.19 (*m*, 4H). ^13^C-NMR (126 MHz, CDCl_3_) *δ* (ppm): 19.53, 20.54, 25.30, 28.31, 39.32, 45.67, 46.91, 48.80, 51.27, 58.44, 113.07, 114.48, 117.28, 118.31, 129.23, 129.48, 148.13, 149.17.

#### 3.1.7. Synthesis of (7*R*,8a*R*)-9,9-Dimethyl-1,3-diphenyl-3,5,6,7,8,8a-hexahydro-4*H*-4a,7-methanoquinazolin-1-ium Chloride (**7a**)

A mixture of diamine **6a** (0.25 mmol, 80 mg, *dr* 99:1), triethyl orthoformate (1.5 mL), and NH_4_Cl (0.26 mmol, 13 mg) was stirred for 5 h at 120 °C. The reaction mixture was cooled to room temperature, and then Et_2_O (5 mL) was added. The resulting precipitate was filtered off, and the filter cake was washed thoroughly with CH_2_Cl_2_. The filtrate was dried over anhydrous Na_2_SO_4_, filtered, and the volatiles were evaporated in vacuo. The product **6a** was recrystallized from *i*-PrOH at room temperature via slow evaporation. Yield: 62 mg (0.170 mmol, 68%, *dr* 99:1) of white solid; mp = 143–146 °C. [*α*]_D_^r.t.^ = +3.6 (0.14, CHCl_3_). EI-HRMS: *m*/*z* = 331.2166 (M^+^); C_23_H_27_N_2_ requires: *m*/*z* = 331.2169 (M^+^); *ν*_max_ 3013, 2953, 2933, 2880, 1663, 1592, 1497, 1455, 1381, 1330, 1300, 1240, 1212, 1030, 934, 767, 728, 704, 638 cm^−1^. ^1^H-NMR (500 MHz, CDCl_3_) *δ* (ppm): 1.03 (*s*, 3H), 1.15 (*s*, 3H), 1.30–1.38 (*m*, 1H), 1.66 (*dd*, *J* = 8.6, 13.7 Hz, 1H), 1.70–1.76 (*m*, 1H), 1.80–1.91 (*m*, 4H), 3.61 (*d*, *J* = 13.9 Hz, 1H), 4.83 (*d*, *J* = 13.9 Hz, 1H), 4.93 (*dd*, *J* = 4.8, 8.7 Hz, 1H), 7.39–7.44 (*m*, 2H), 7.45–7.51 (*m*, 4H), 7.57–7.62 (*m*, 2H), 7.64–7.69 (*m*, 2H), 8.06 (*s*, 1H). ^13^C-NMR (126 MHz, CDCl_3_) *δ* (ppm): 20.12, 20.21, 26.58, 32.21, 34.18, 45.66, 46.16, 47.97, 48.61, 60.65, 124.17, 124.70, 129.32, 129.40, 130.20, 130.33, 138.82, 141.81, 152.01.

#### 3.1.8. Synthesis of (7*R*,8a*R*)-9,9-Dimethyl-1,3-diphenyl-3,5,6,7,8,8a-hexahydro-4*H*-4a,7-methanoquinazolin-1-ium Tetrafluoroborate (**7b**)

A mixture of diamine **6a** (0.25 mmol, 80 mg, *dr* 99:1), triethyl orthoformate (1.5 mL), and NH_4_BF_4_ (0.26 mmol, 27 mg) was stirred for 5 h at 120 °C. The reaction mixture was cooled to room temperature, and then Et_2_O (5 mL) was added. The resulting precipitate was filtered off, and the filter cake was washed thoroughly with CH_2_Cl_2_. The filtrate was dried over anhydrous Na_2_SO_4_, filtered, and the volatiles were evaporated in vacuo. Product **6a** was recrystallized from *i*-PrOH at room temperature by slow evaporation. Yield: 75 mg (0.180 mmol, 72%, *dr* 99:1) of grayish white solid; mp = 280–285 °C. [*α*]_D_^r.t.^ = +30.8 (0.25, CHCl_3_). EI-HRMS: *m*/*z* = 331.2170 (M^+^); C_23_H_27_N_2_ requires: *m*/*z* = 331.2169 (M^+^); *ν*_max_ 2956, 2921, 1663, 1591, 1494, 1380, 1319, 1297, 1229, 1049, 1030, 916, 766, 696 cm^−1^. ^1^H-NMR (500 MHz, CDCl_3_) *δ* (ppm): 1.04 (*s*, 3H), 1.13 (*s*, 3H), 1.31–1.38 (*m*, 1H), 1.64–1.92 (*m*, 6H), 3.61 (*d*, *J* = 14.3 Hz, 1H), 4.46 (*d*, *J* = 14.3 Hz, 1H), 4.55 (*dd*, *J* = 5.3, 8.1 Hz, 1H), 7.37–7.53 (*m*, 10H), 7.85 (*s*, 1H). ^13^C-NMR (126 MHz, CDCl_3_) *δ* (ppm): 20.22, 20.25, 26.56, 32.24, 34.35, 45.65, 46.12, 47.98, 48.05, 60.03, 123.99, 124.42, 129.52, 129.67, 130.42, 130.55, 138.69, 141.72, 151.62.

#### 3.1.9. Synthesis of (4a*S*,7*R*)-9,9-Dimethyl-1,3-diphenyl-3,5,6,7,8,8a-hexahydro-4*H*-4a,7-methanoquinazolin-1-ium Tetrafluoroborate (**7c**)

A mixture of diamine **6a** (0.25 mmol, 80 mg, *dr* 90:10), triethyl orthoformate (1.5 mL), and NH_4_BF_4_ (0.26 mmol, 27 mg) was stirred for 5 h at 120 °C. The reaction mixture was cooled to room temperature, and then Et_2_O (5 mL) was added. The resulting precipitate was filtered off, and the filter cake was washed thoroughly with CH_2_Cl_2_. The filtrate was dried over anhydrous Na_2_SO_4_, filtered, and the volatiles were evaporated in vacuo. Product **6a** was recrystallized from *i*-PrOH at room temperature by slow evaporation. Yield: 68 mg (0.1625 mmol, 65%, *dr* 91:9) of grayish white solid; mp = 238–240 °C. [*α*]_D_^r.t.^ = –25.5 (0.13, CHCl_3_). EI-HRMS: *m*/*z* = 331.2163 (M^+^); C_23_H_27_N_2_ requires: *m*/*z* = 331.2169 (M^+^); *ν*_max_ 3082, 3064, 2952, 2881, 1645, 1591, 1494, 1457, 1419, 1396, 1368, 1330, 1285, 1232, 1162, 1050, 1026, 968, 829, 770, 753, 697, 656, 627 cm^−1^. ^1^H-NMR (500 MHz, CDCl_3_) *δ* (ppm): 0.97 (*dd*, *J* = 5.9, 13.8 Hz, 1H), 1.08 (*s*, 3H), 1.20 (*s*, 3H), 1.23–1.29 (*m*, 1H), 1.69–1.78 (*m*, 1H), 1.87–2.00 (*m*, 3H), 2.19–2.28 (*m*, 1H), 3.49 (*d*, *J* = 12.8 Hz, 1H), 4.44 (*d*, *J* = 12.8 Hz, 1H), 4.91 (*dd*, *J* = 6.0, 11.1 Hz, 1H), 7.36–7.54 (*m*, 10H), 7.83 (*s*, 1H). ^13^C-NMR (126 MHz, CDCl_3_) *δ* (ppm): 18.49, 20.07, 27.41, 28.05, 30.79, 45.49, 46.37, 47.61, 54.91, 59.40, 124.54, 124.78, 129.49, 129.70, 130.29, 130.41, 139.33, 141.95, 152.66.

#### 3.1.10. Synthesis of *N*-(((1*S*,2*S*,4*R*)-7,7-Dimethyl-2-(phenylamino)bicyclo[2.2.1]heptan-1-yl)methyl)-*N*-phenylformamide (**8**) and *N*-(((1*S*,2*R*,4*R*)-7,7-Dimethyl-2-(phenylamino)bicyclo[2.2.1]heptan-1-yl)methyl)-*N*-phenylformamide (**8**′)

To a solution of ***ent*-7c** (0.0239 mmol, 10 mg, *dr* 82:18) in THF (1 mL), H_2_O (100 μL) and NaOH (0.0478 mmol, 1.9 mg) was added. The resulting reaction mixture was stirred for 18 h at room temperature. The reaction mixture was diluted with Et_2_O (20 mL) and washed with H_2_O (1 mL) and brine (1 mL). The organic phase was dried over anhydrous Na_2_SO_4_, filtered, and the volatiles were evaporated in vacuo. Yield: 6 mg (0.0172 mmol, 72%, *dr* 81:19) of colorless semisolid. ^1^H-NMR (500 MHz, CDCl_3_) for **8**′: *δ* (ppm): 0.73–0.79 (*m*, 1H), 0.93 (*s*, 3H), 3.04 (*dd*, *J* = 4.7, 7.3, 1H), 3.61 (*d*, *J* = 11.1, 1H), 4.64 (*d*, *J* = 14.9, 1H), 4.81 (*s*, 1H), 6.51–6.56 (*m*, 2H), 8.17 (s, 1H). ^13^C-NMR (126 MHz, CDCl_3_) for **8′**: *δ* (ppm): 20.93, 21.01, 30.45, 35.33, 41.41, 43.11, 45.69, 48.85, 53.55, 59.23, 113.14, 116.55, 125.27, 127.41, 129.13, 129.64, 141.60, 147.75, 163.82.

The crude product was additionally crystallized from a mixture of CHCl_3_ and *n*-heptane via the slow evaporation of chloroform at room temperature. Compound **8**: Yield: 3 mg (0.0086 mmol, 36%, *dr* 95:5) of white solid; mp = 144–146 °C. EI-HRMS: *m*/*z* = 349.2275 (M^+^); C_23_H_29_N_2_O requires: *m*/*z* = 349.2274 (M^+^); *ν*_max_ 3383, 2965, 2943, 2863, 1660, 1592, 1517, 1495, 1432, 1390, 1358, 1313, 1257, 1215, 1180, 1128, 1069, 1022, 988, 917, 866, 826, 748, 691, 670 cm^−1^. ^1^H-NMR (500 MHz, CDCl_3_) for **8**: *δ* (ppm): 0.89 (*s*, 3H), 0.87–0.93 (*m*, 1H), 1.03 (*s*, 3H), 1.14–1.21 (*m*, 1H), 1.44–1.52 (*m*, 1H), 1.61–1.77 (*m*, 3H), 2.29–2.38 (*m*, 1H), 3.63 (*d*, *J* = 9.0, 1H), 3.87 (*d*, *J* = 14.8, 1H), 4.07 (*s*, 1H), 4.14 (*d*, *J* = 14.8, 1H), 6.43–6.48 (*m*, 2H), 6.66 (*t*, *J* = 7.3, 1H), 7.09–7.16 (*m*, 4H), 7.17–7.21 (*m*, 1H), 7.29–7.36 (*m*, 2H), 8.31 (*s*, 1H). ^13^C-NMR (126 MHz, CDCl_3_) for **8**: *δ* (ppm): 19.31, 20.14, 25.48, 27.95, 38.60, 44.43, 45.84, 48.67, 53.67, 56.60, 113.50, 117.18, 123.62, 126.57, 129.22, 129.71, 142.15, 148.49, 163.72.

### 3.2. General Procedure for the Catalytic Asymmetric Benzoin Condensation Reaction with Benzaldehyde

To a solution/suspension of amidinium salt 7 (10 mol%) in anhydrous THF or 1,4-dioxane (in the case of Na_2_CO_3_, water was used as solvent), benzaldehyde (0.75 mmol) and then a base (10 mol%; *t*BuONa, LiHDMS, and LDA (1 M in THF/hexanes)) were added. The resulting reaction mixture was stirred for 24 h at room temperature. The reaction mixture was concentrated under reduced pressure. Part of the residue was used for ^1^H-NMR measurements, and the rest was subjected to column chromatography.

### 3.3. X-ray Crystallography

Single-crystal X-ray diffraction data were collected with an Agilent Technologies SuperNova Dual diffractometer with an Atlas detector using monochromatic Cu-Kα radiation (λ = 1.54184 Å) at 150 K. CrysAlis PRO was used to process the data [37]. Olex2.1.2 [38] was used to solve the structures using the direct methods implemented in SHELXS [39] or SHELXT [40] and refined using a full matrix least squares method based on F2 and SHELXT-2014/7 [41]. All non-hydrogen atoms were refined anisotropically. The hydrogen atoms were placed at geometrically calculated positions and refined with a riding model. Mercury [42] and Platon [43] were used for the drawings and the analysis of bond lengths, bond angles, and intermolecular interactions. Structural and other crystallographic details for data collection and the refinement of compounds **4**, **7a,** and **8** were deposited with the Cambridge Crystallographic Data Centre under CCDC Deposition Numbers 2302865, 2302861, and 2307379, respectively. These data are available free of charge at https://www.ccdc.cam.ac.uk/structures/, accessed on 13 November 2023 (or from CCDC, 12 Union Road, Cambridge CB2 1EZ, UK; fax: +44 1223 336033; e-mail: deposit@ccdc.cam.ac.uk).

## 4. Conclusions

Starting from (1*S*)-(+)-ketopinic acid, three NHC precursors based on camphor were prepared in a diastereoselective five-step synthesis. Both the *exo*- and *endo*-diastereomer of the NHC precursors were also analyzed in an asymmetric benzoin reaction. The desired benzoin product was not observed under any reaction conditions. On the other hand, we showed that amidinium salt procatalysts **7** decompose under basic conditions. The hydrolytic instability can be attributed to the non-aromatic nature of the amidinium salts studied compared to the triazolium salts shown in Figure 1. All new compounds were fully characterized, including the determination of the absolute configuration by X-ray diffraction, NOESY measurements, and NMR data correlation.

## Data Availability

Data are contained within the article and Supplementary Materials.

## Supplementary Materials

The following supporting information can be downloaded at https://www.mdpi.com/article/10.3390/molecules28247973/s1: Copies of ^1^H- and ^13^C-NMR spectra; Copies of NOESY spectra; Copies of HRMS reports; Copies of IR spectra, Structure determination using X-ray diffraction analysis.
