# Supplementary material for: Synthesis of Six-Membered N-Heterocyclic Carbene Precursors Based on Camphor
Source: Molecules. 2023 Dec 6;28(24):7973. doi: 10.3390/molecules28247973 (PMC10745536; doi:10.3390/molecules28247973)
Supplement: Supplementary file 1 [file molecules-28-07973-s001.zip › molecules-2743611-supplementary.pdf]

Supporting Information

Synthesis of Six-membered N-Heterocyclic Carbene Precursors Based on Camphor

Jan Šegina, Luka Ciber, Helena Brodnik, Franc Požgan, Jurij Svete, Bogdan Štefane, and Uroš Grošelj*

University of Ljubljana, Faculty of Chemistry and Chemical Technology, Večna pot 113, 1000 Ljubljana, Slovenia

* Correspondence: uros.groselj@fkkt.uni-lj.si; Tel.: +386-1-479-8565 (U.G.)

Table of contents

1. NMR spectra 2
2. IR spectra 11
3. MS spectra 19
4. Structure Determination by NMR 27
5. Structure determination by X-ray diffraction analysis 31

1. NMR spectra

(1*R*,4*R*,*E*)-7,7-Dimethyl-*N*-phenyl-2-(phenylimino)bicyclo[2.2.1]heptane-1-carboxamide (4)

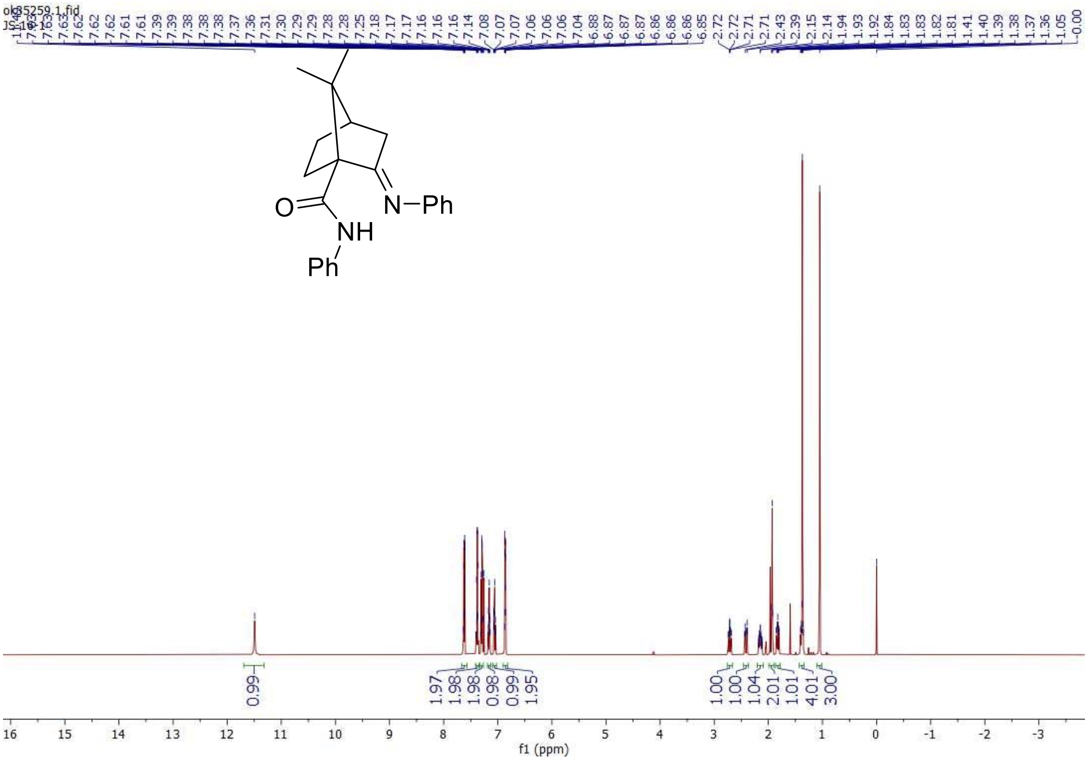

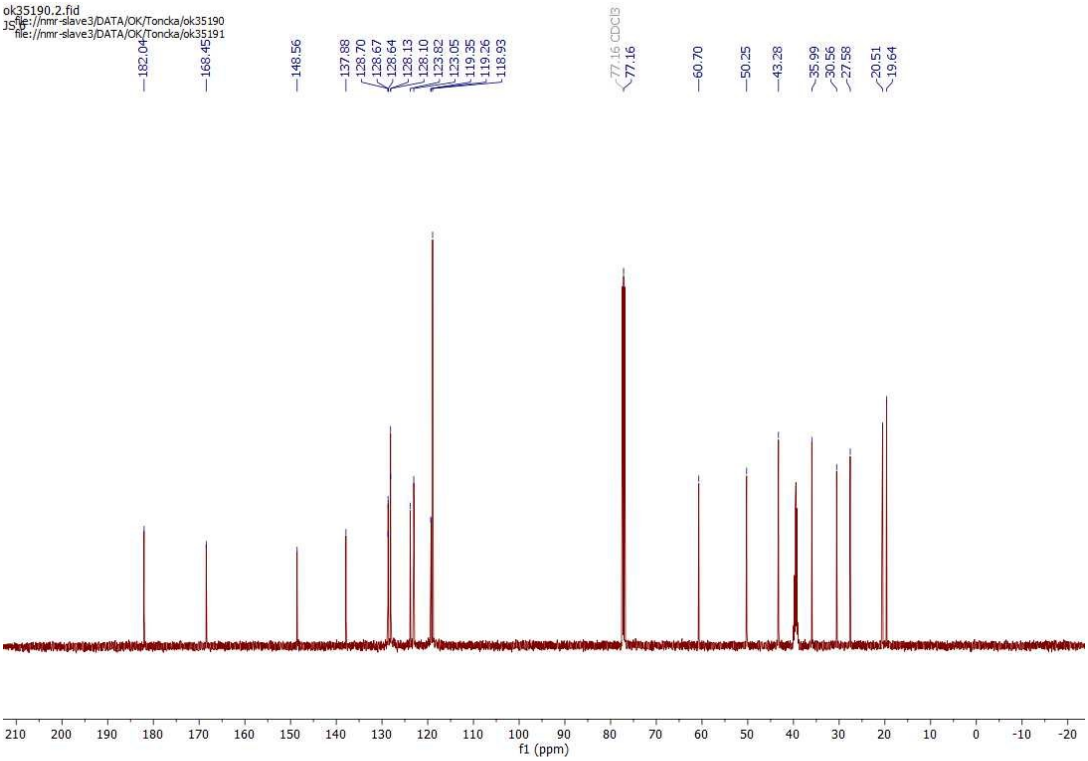

(1*R*,2*R*,4*R*)-7,7-Dimethyl-*N*-phenyl-2-(phenylamino)bicyclo[2.2.1]heptane-1-carboxamide (5a)

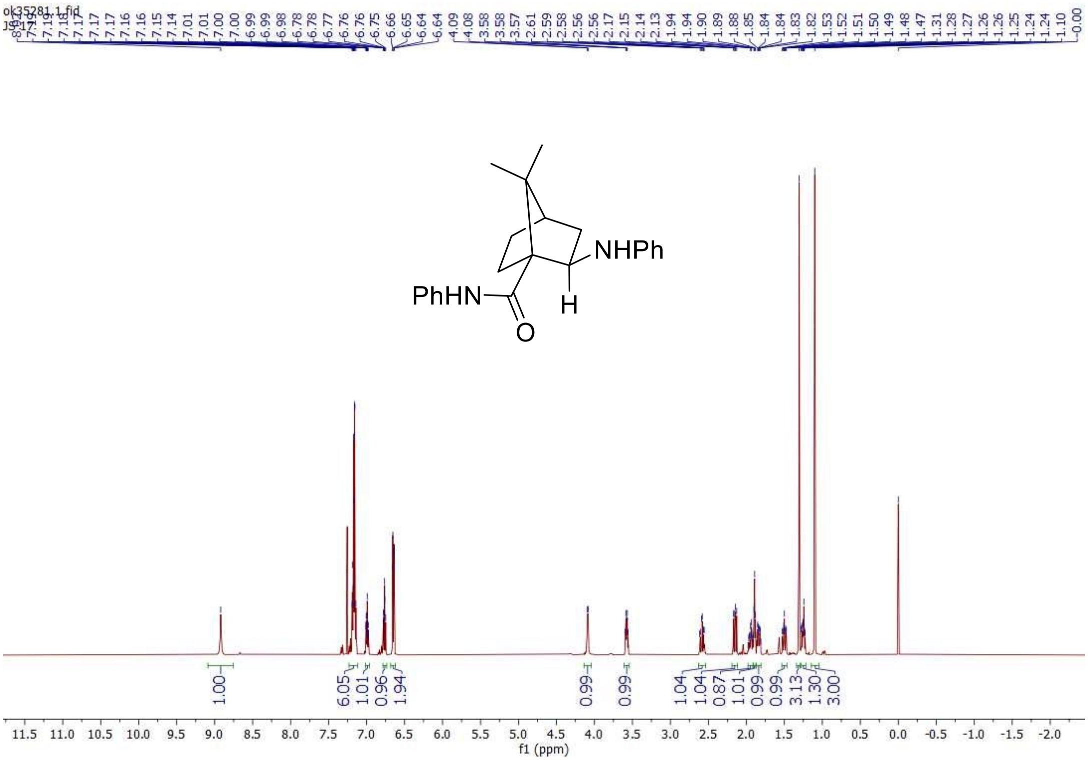

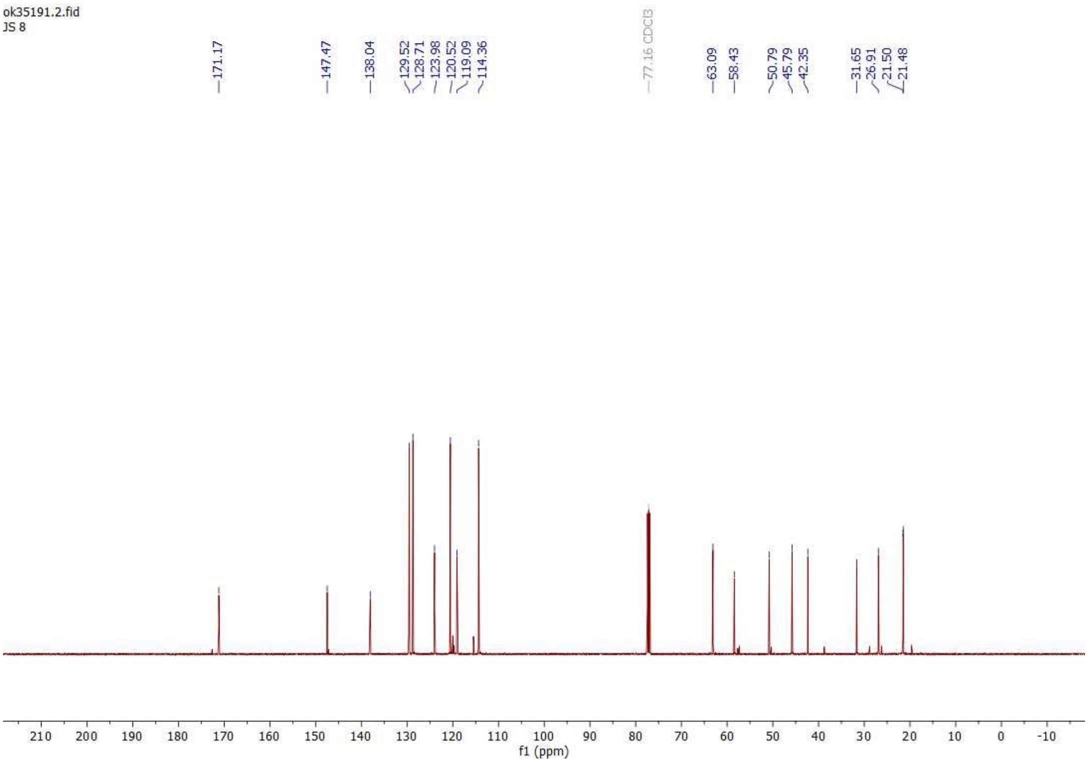

(1*S*,2*R*,4*R*)-7,7-Dimethyl-*N*-phenyl-1-((phenylamino)methyl)bicyclo[2.2.1]heptan-2-amine (6a)

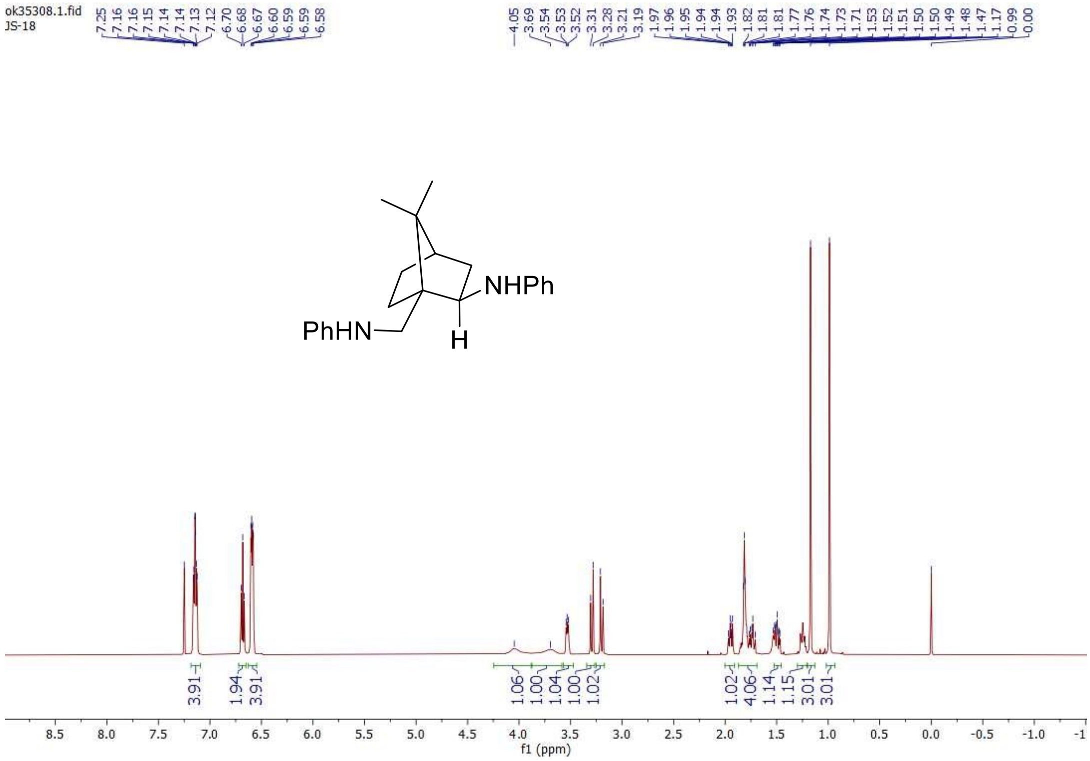

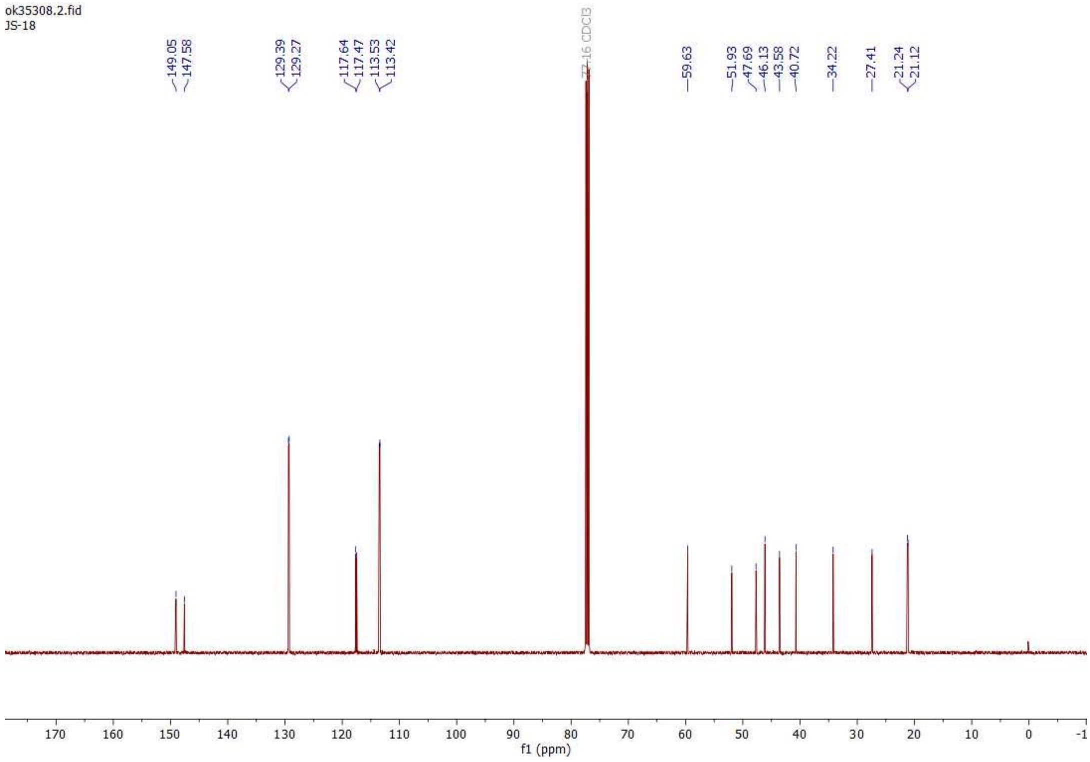

(1*S*,2*S*,4*R*)-7,7-Dimethyl-*N*-phenyl-1-((phenylamino)methyl)bicyclo[2.2.1] heptan-2-amine (6b)

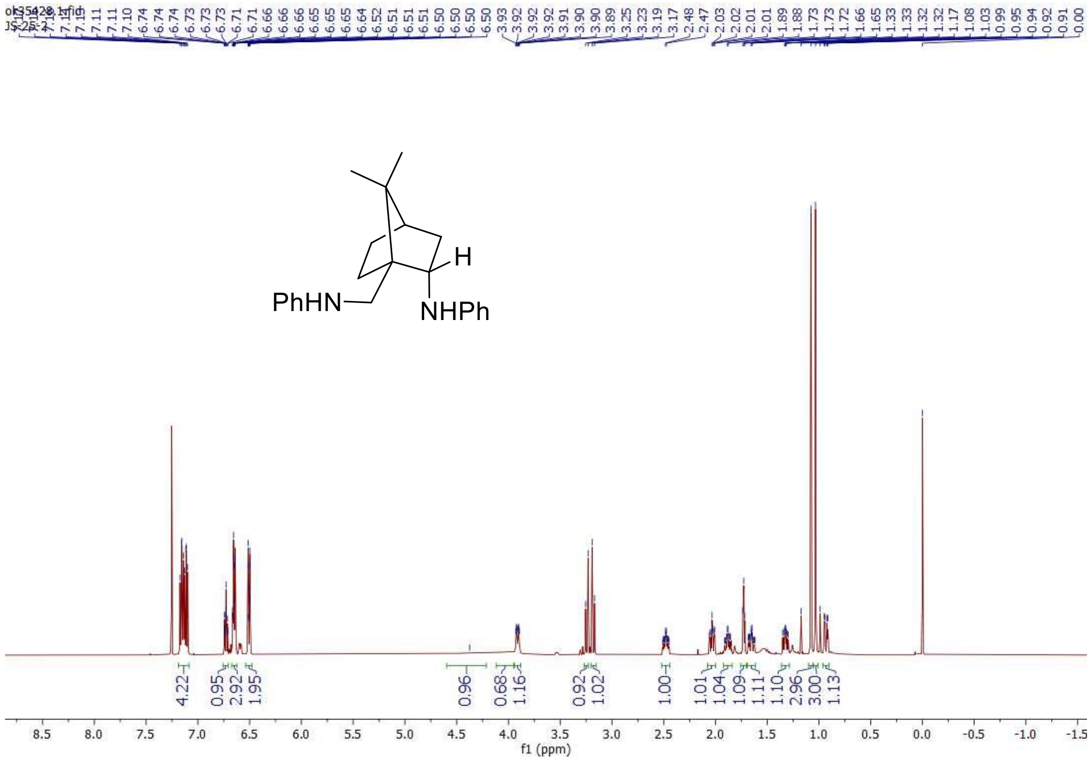

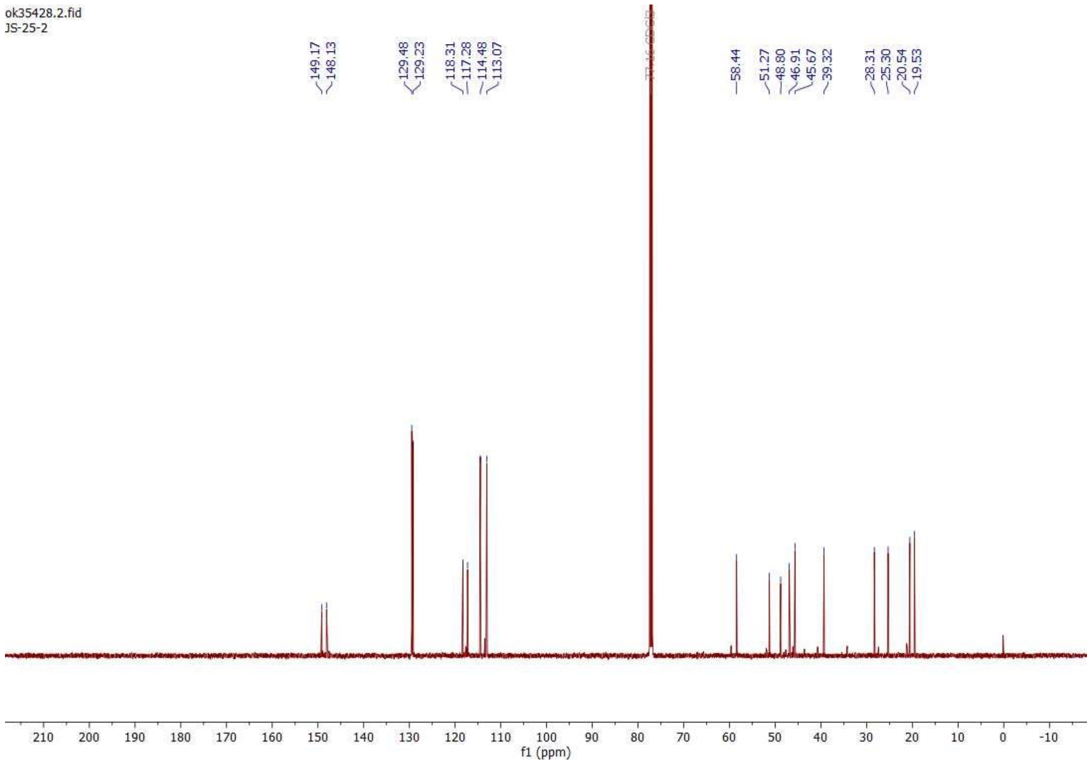

(7*R*,8a*R*)-9,9-Dimethyl-1,3-diphenyl-3,5,6,7,8,8a-hexahydro-4*H*-4a,7-methanoquinazolin-1-ium chloride (7a)

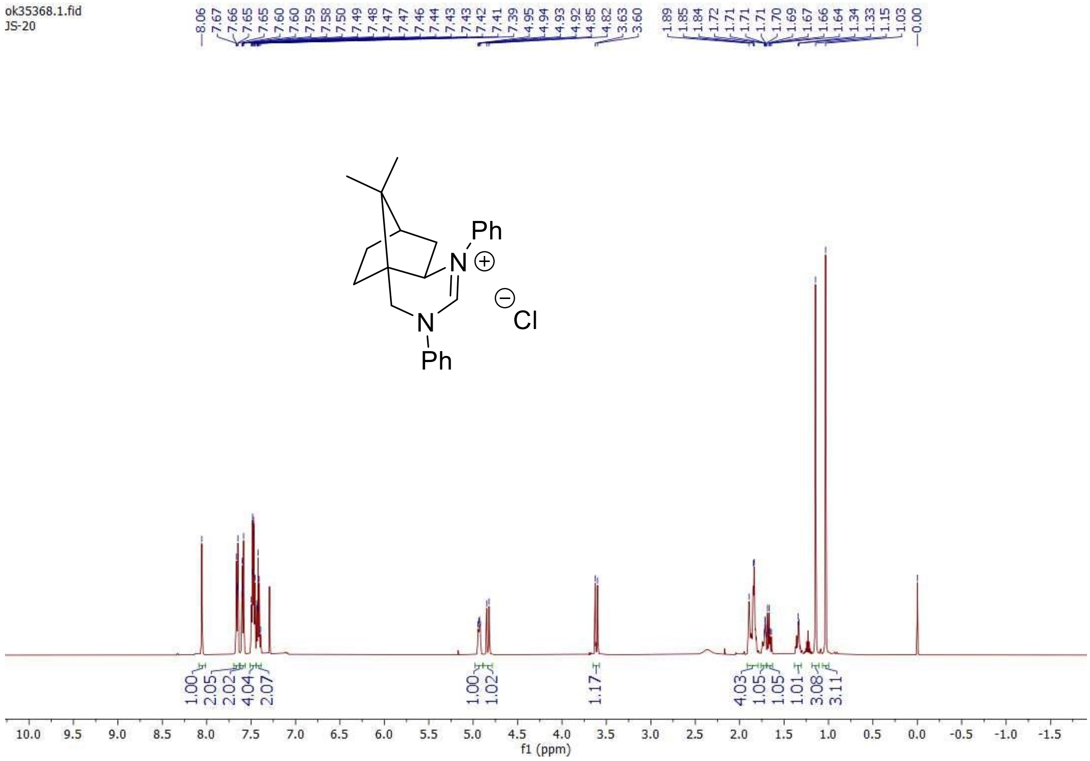

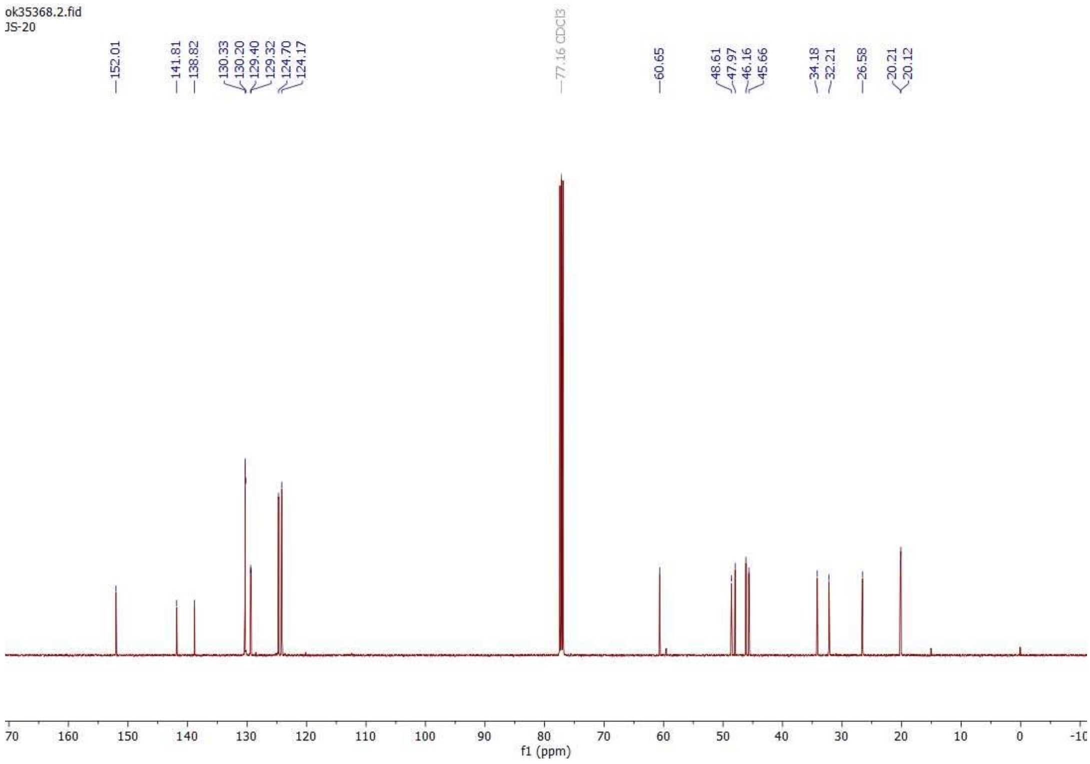

(7*R*,8a*R*)-9,9-Dimethyl-1,3-diphenyl-3,5,6,7,8,8a-hexahydro-4*H*-4a,7-methanoquinazolin-1-ium tetrafluoroborate (7b)

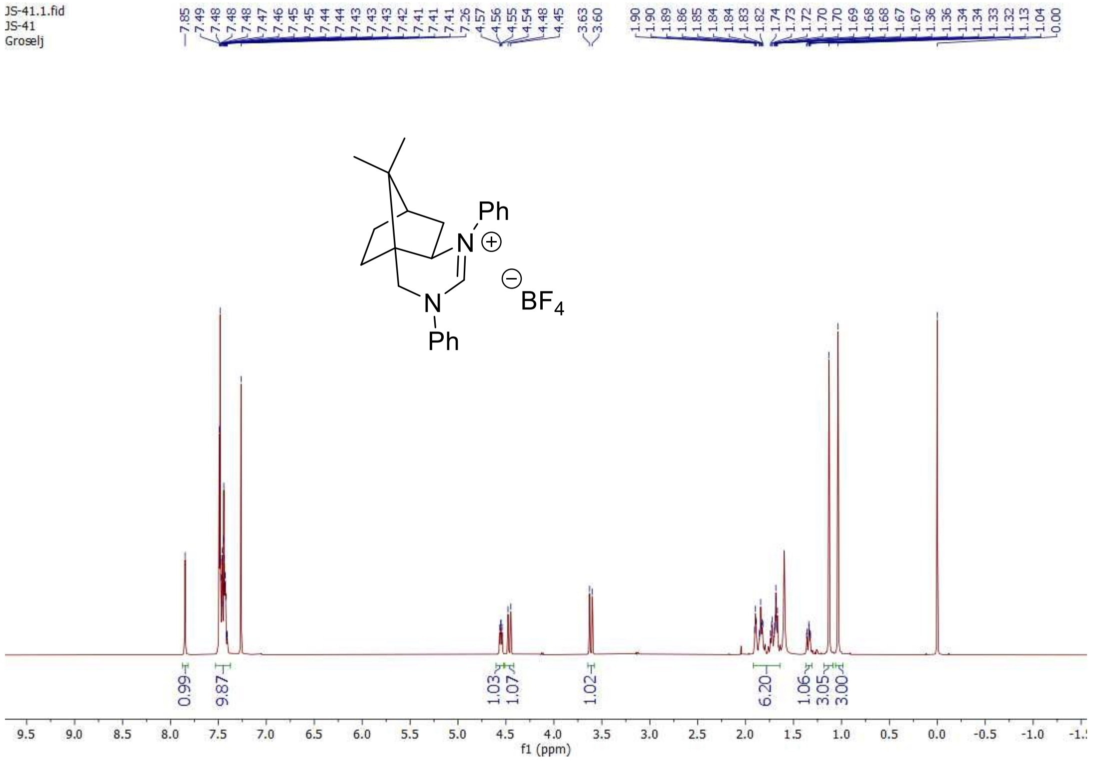

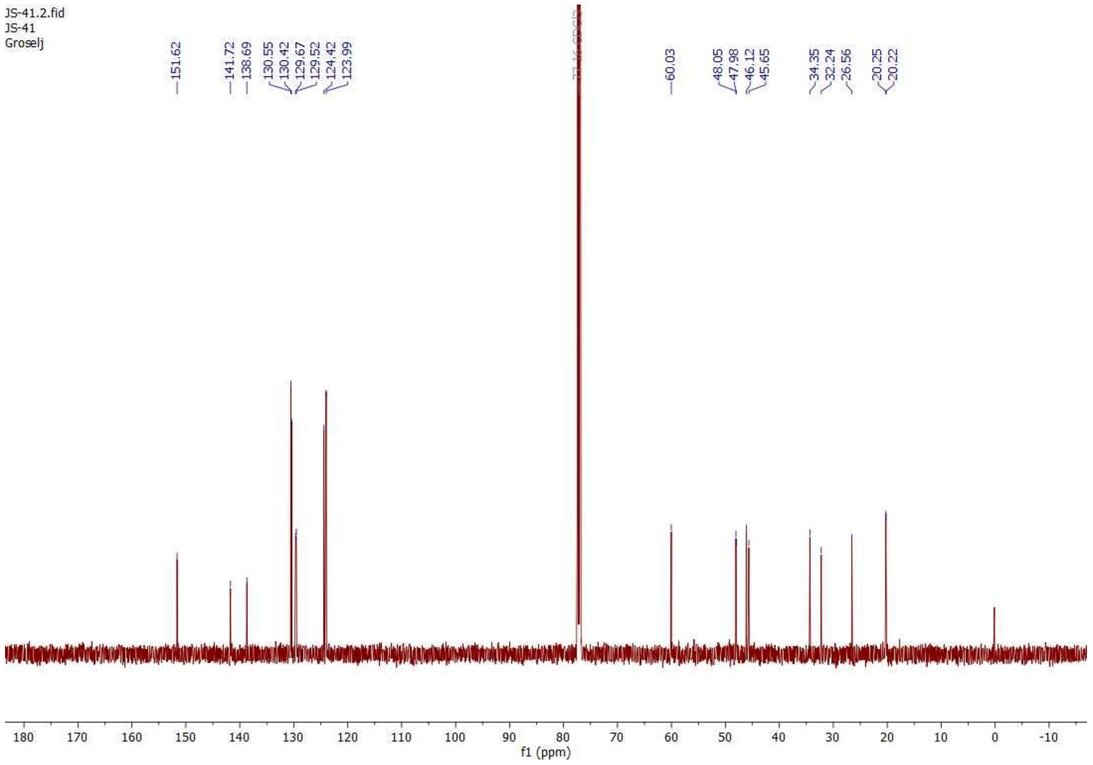

(4a*S*,7*R*)-9,9-Dimethyl-1,3-diphenyl-3,5,6,7,8,8a-hexahydro-4*H*-4a,7-methanoquinazolin-1-ium tetrafluoroborate (7c)

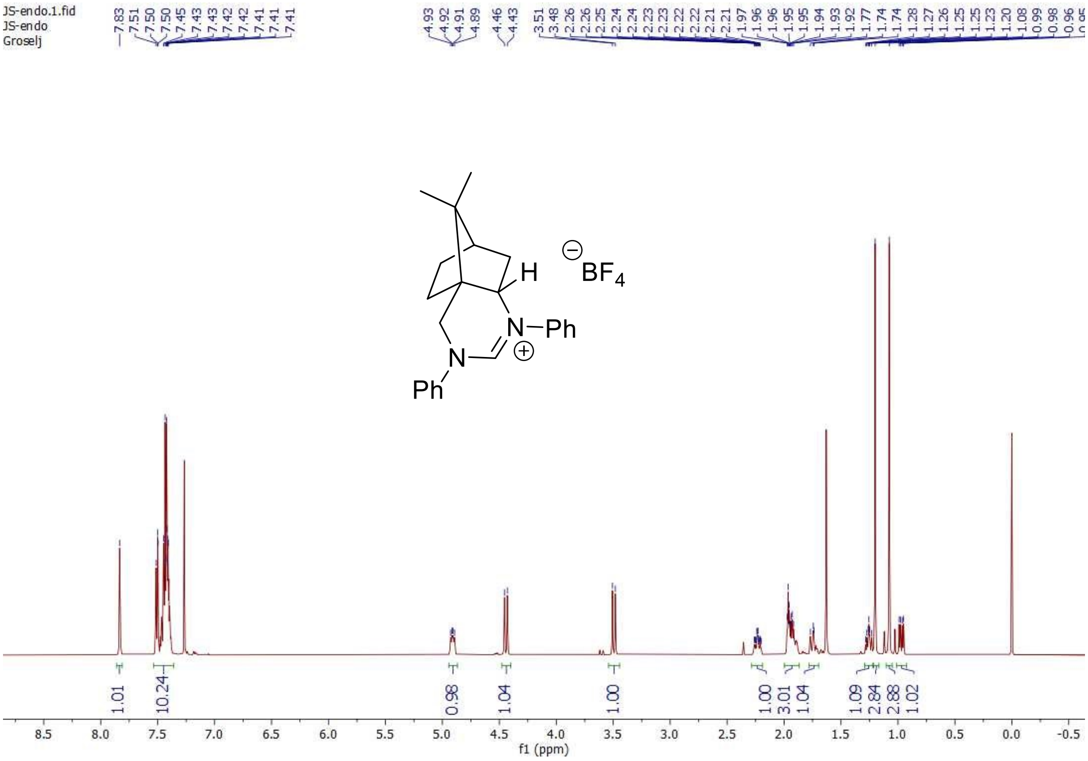

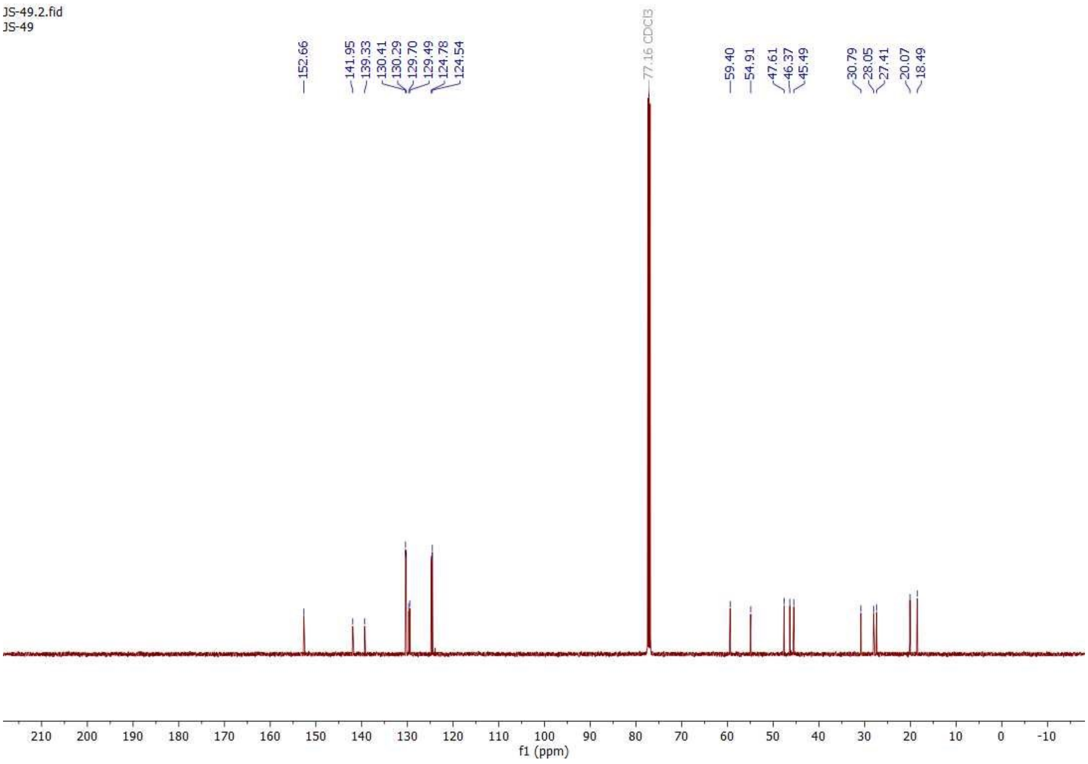

***N*-(((1*R*,2*R*,4*S*)-7,7-Dimethyl-2-(phenylamino)bicyclo[2.2.1]heptan-1-yl)methyl)-*N*-phenylformamide (8) and *N*-(((1*R*,2*S*,4*S*)-7,7-dimethyl-2-(phenylamino)bicyclo[2.2.1]heptan-1-yl)methyl)-*N*-phenylformamide (8’)**

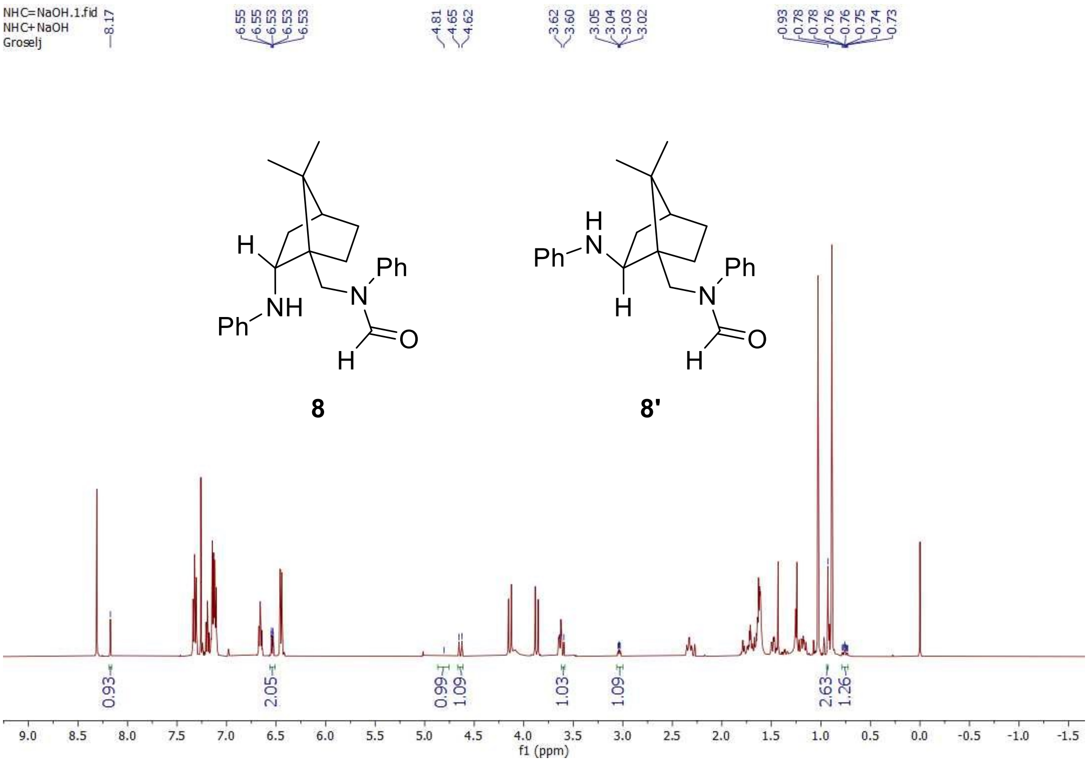

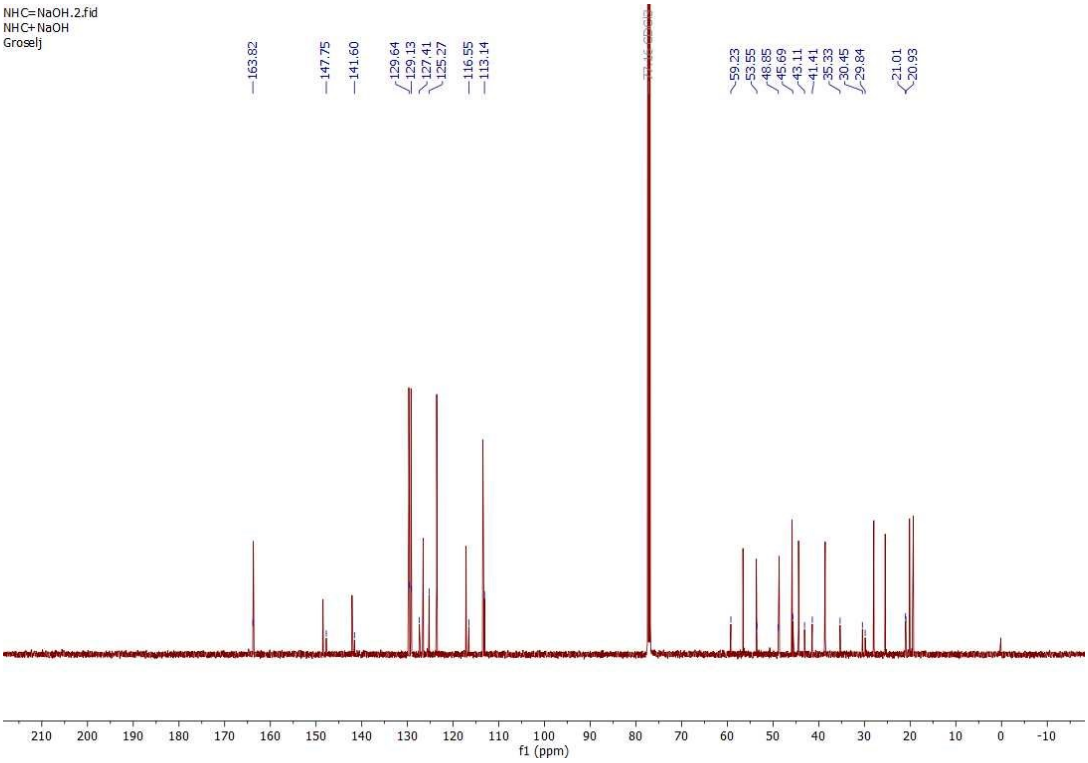

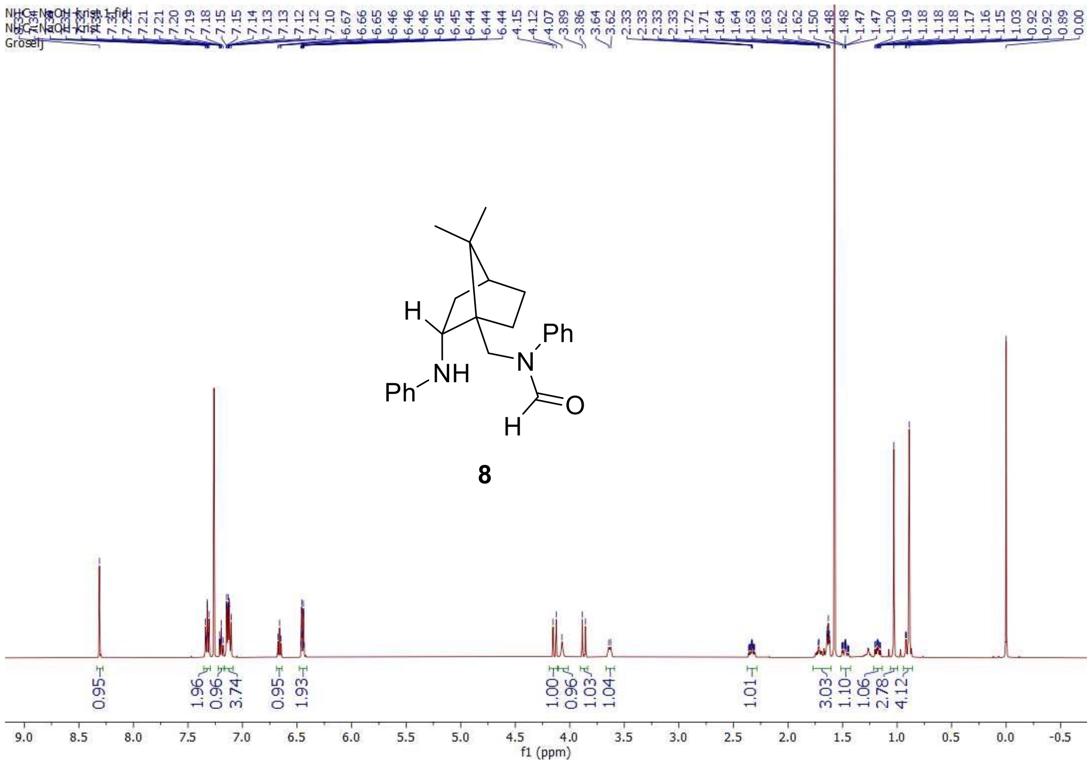
NHC=NaOH-krist.1.fid
NHC=NaOH-krist
Groselj
8
0.95
1.96
0.96
3.74
0.95
1.93
1.00
0.96
1.03
1.04
1.01
3.03
1.10
1.06
2.78
4.12
f1 (ppm)

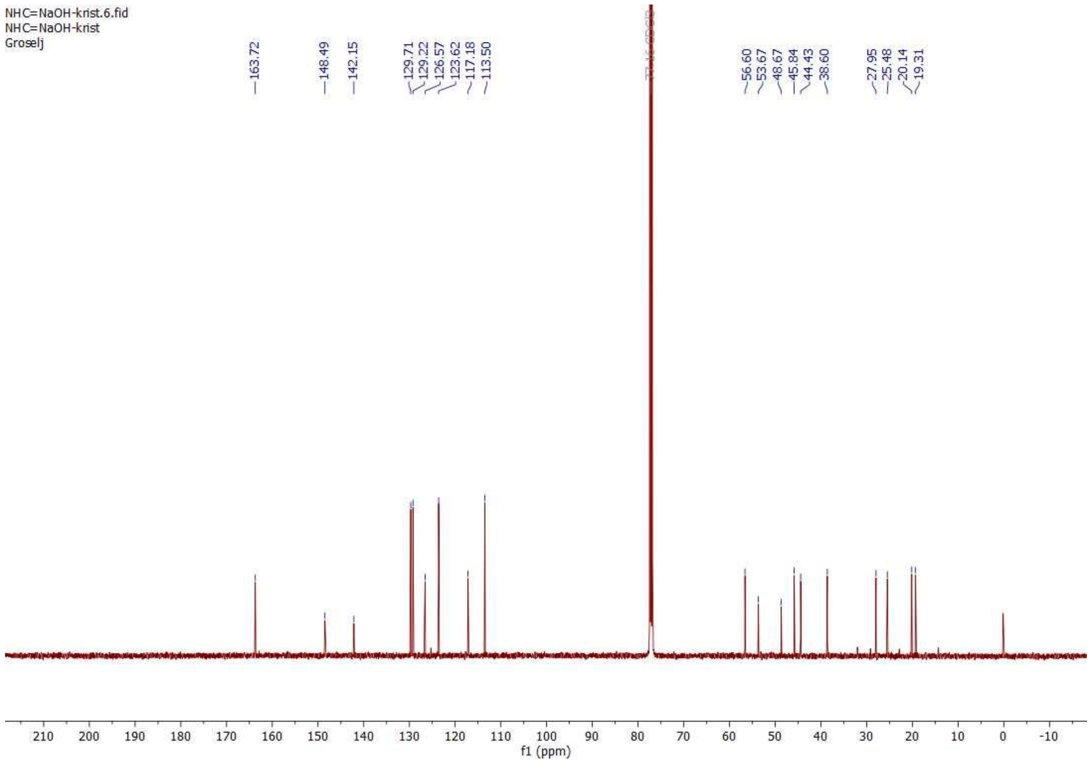
NHC=NaOH-krist.6.fid
NHC=NaOH-krist
Groselj
163.72
148.49
142.15
129.71
129.22
126.57
123.62
117.18
113.50
56.60
53.67
48.67
45.84
44.43
38.60
27.95
25.48
20.14
19.31
f1 (ppm)

2. IR spectra

(1*R*,4*R*,*E*)-7,7-Dimethyl-*N*-phenyl-2-(phenylimino)bicyclo[2.2.1]heptane-1-carboxamide (4)

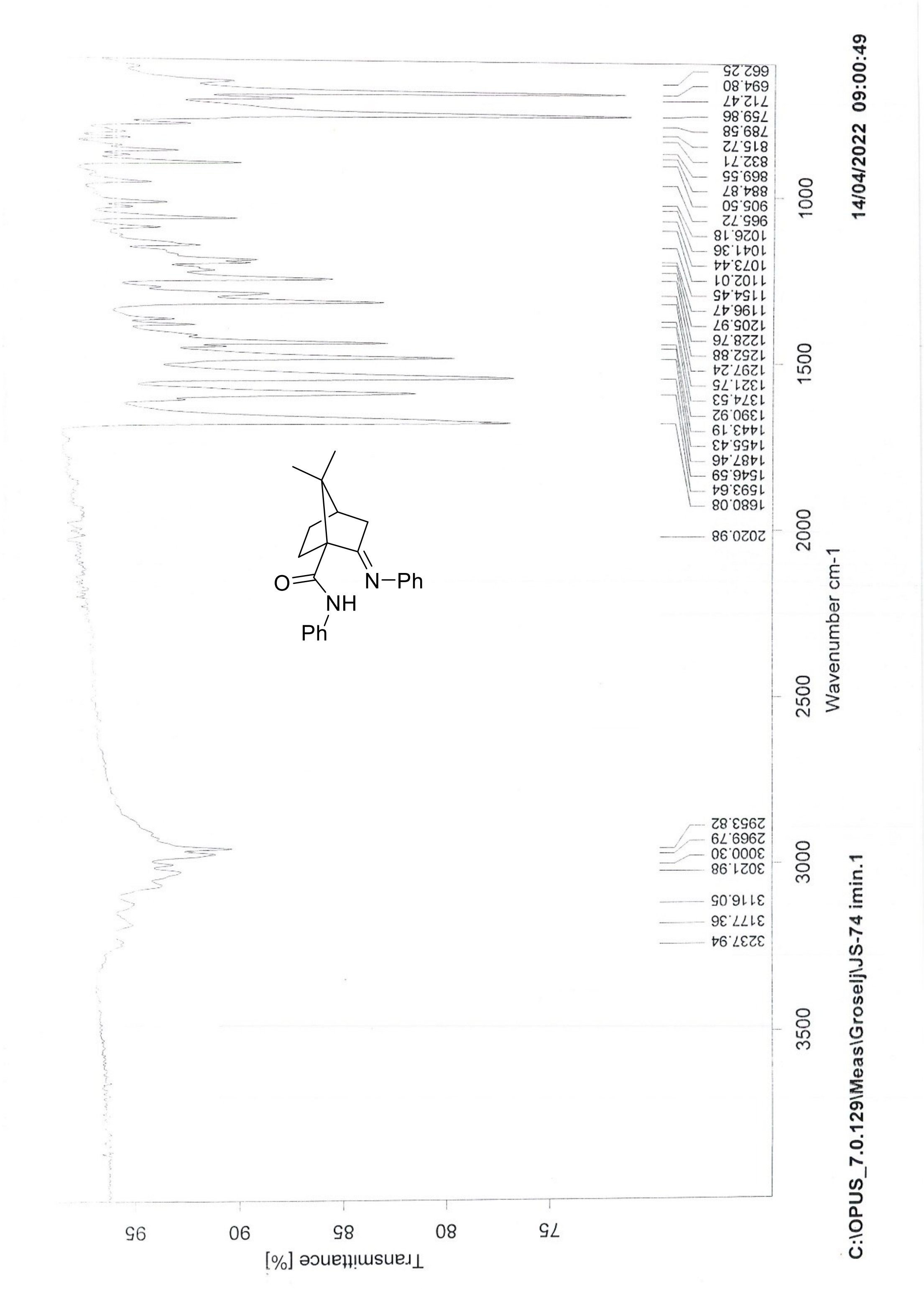

(1*R*,2*R*,4*R*)-7,7-Dimethyl-*N*-phenyl-2-(phenylamino)bicyclo[2.2.1]heptane-1-carboxamide (5a)

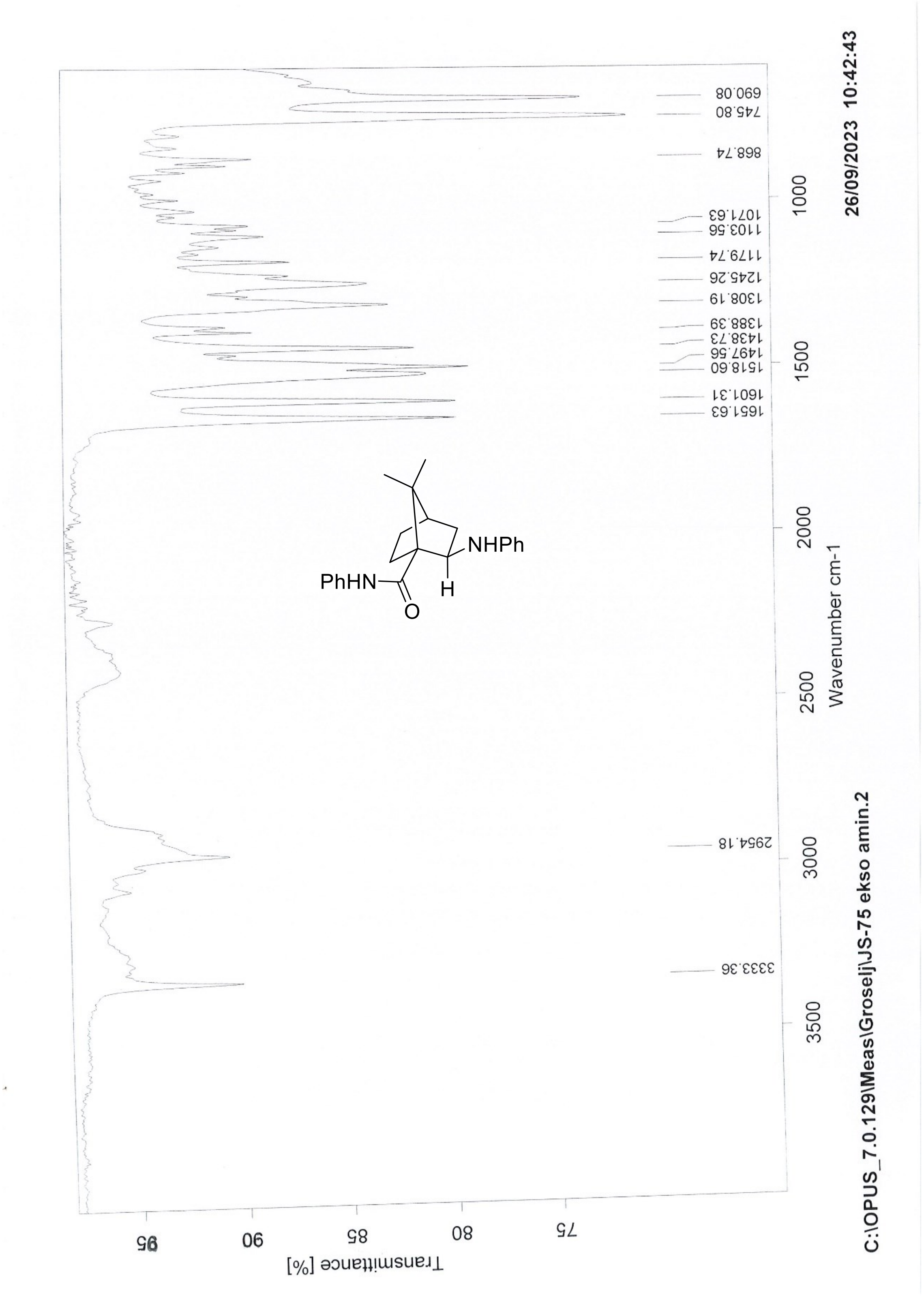

(1*S*,2*R*,4*R*)-7,7-Dimethyl-*N*-phenyl-1-((phenylamino)methyl)bicyclo[2.2.1]heptan-2-amine (6a)

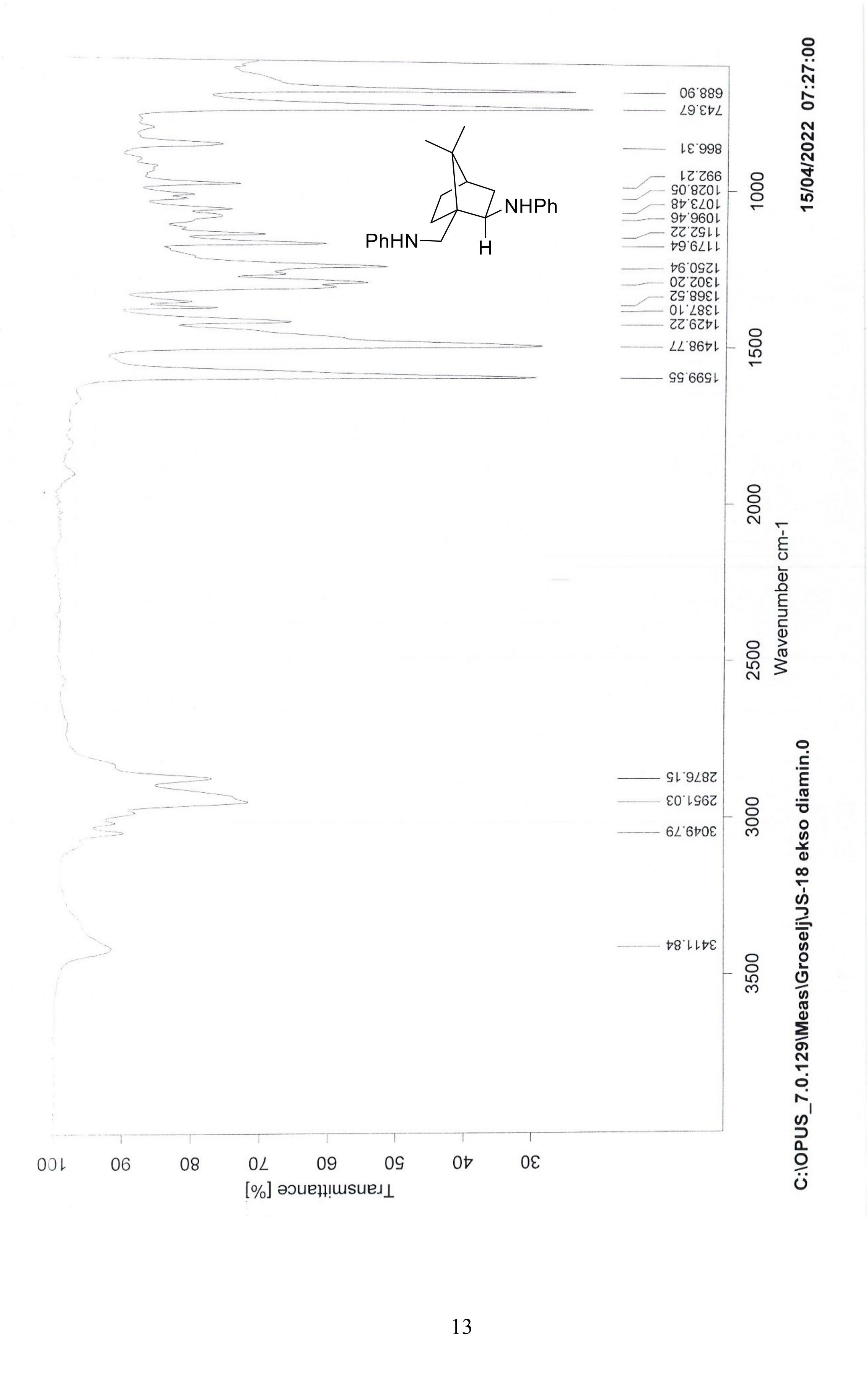

(1*S*,2*S*,4*R*)-7,7-Dimethyl-*N*-phenyl-1-((phenylamino)methyl)bicyclo[2.2.1] heptan-2-amine (6b)

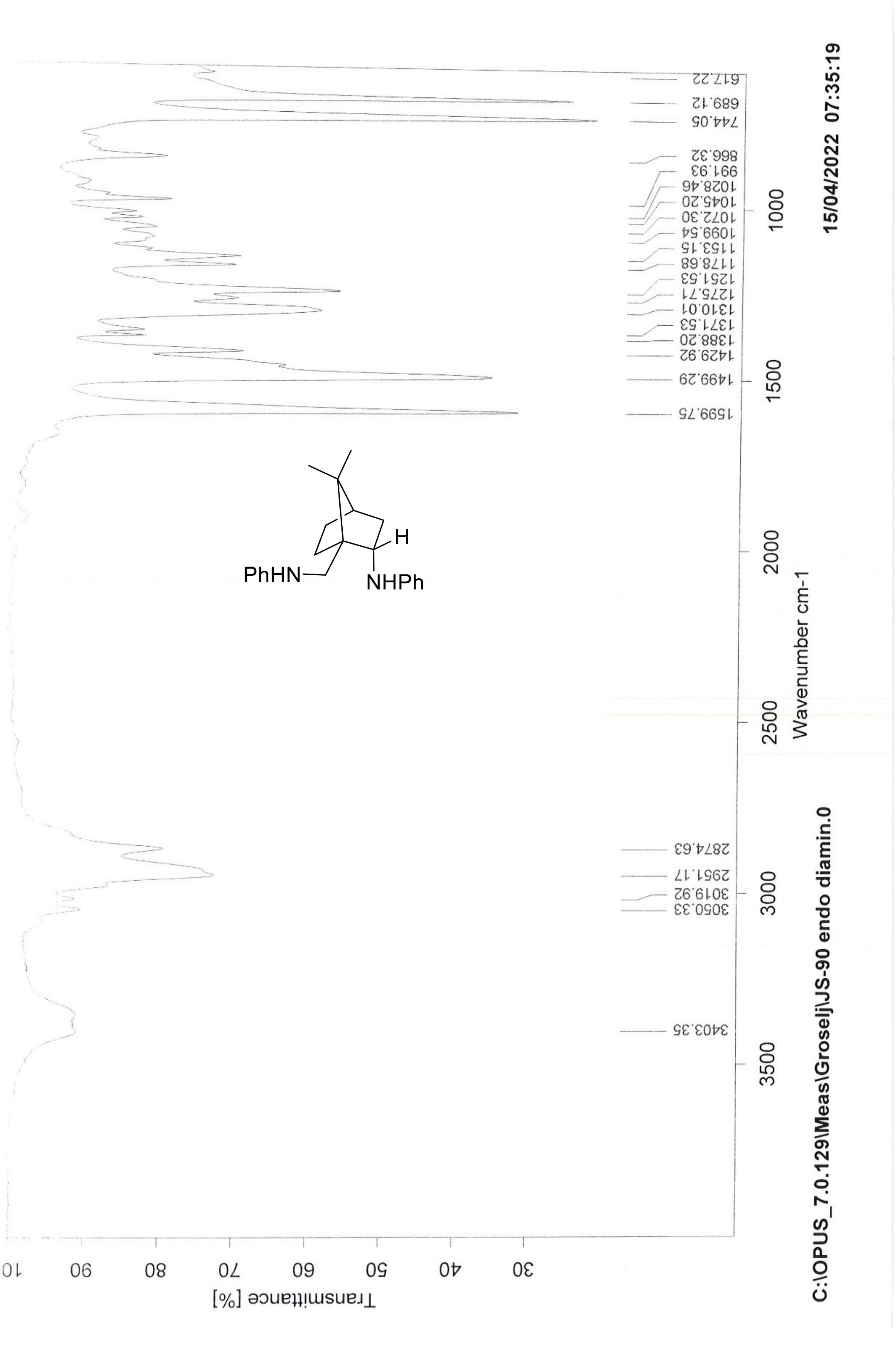

(7*R*,8a*R*)-9,9-Dimethyl-1,3-diphenyl-3,5,6,7,8,8a-hexahydro-4*H*-4a,7-methanoquinazolin-1-ium chloride (7a)

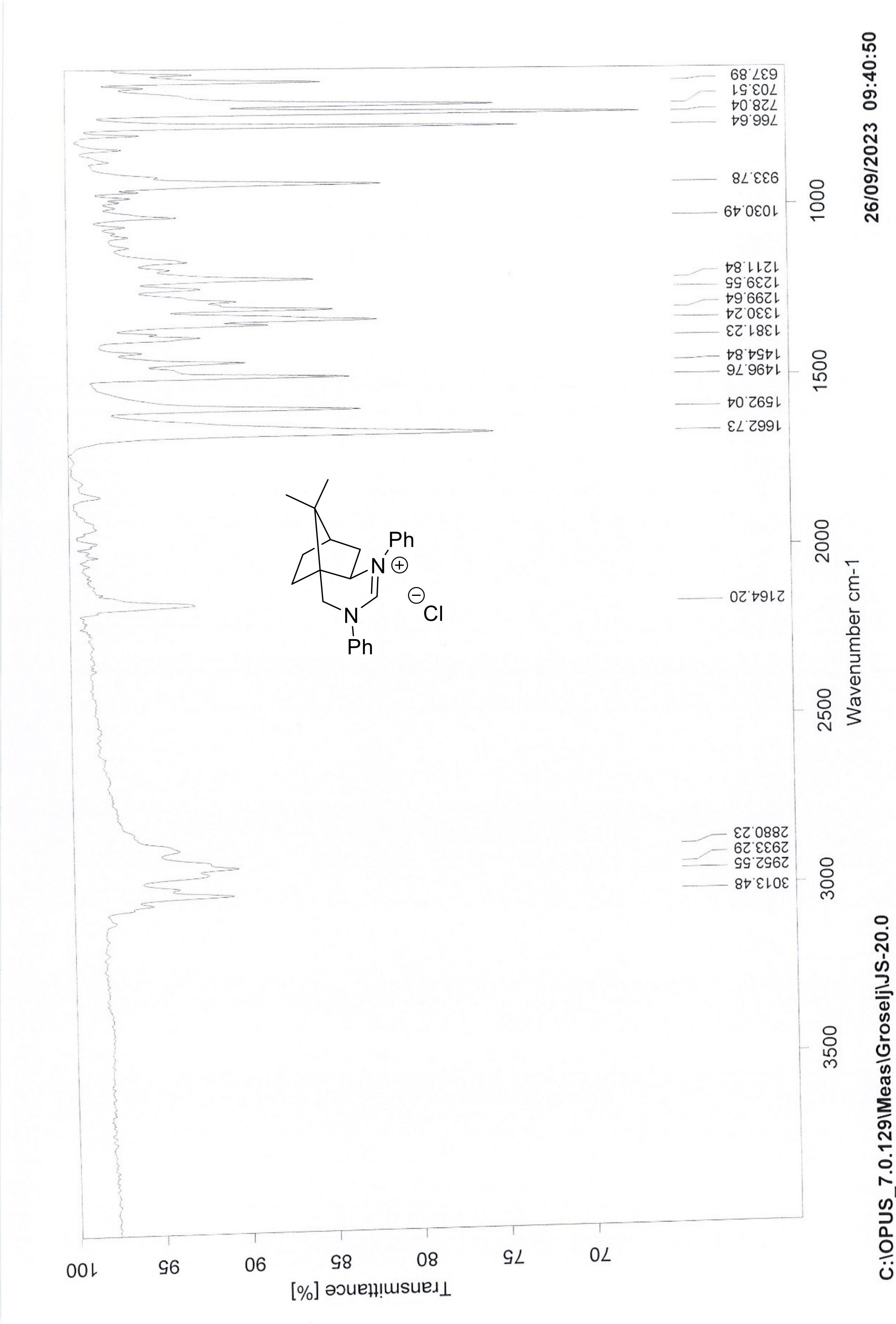

(7*R*,8a*R*)-9,9-Dimethyl-1,3-diphenyl-3,5,6,7,8,8a-hexahydro-4*H*-4a,7-methanoquinazolin-1-ium tetrafluoroborate (7b)

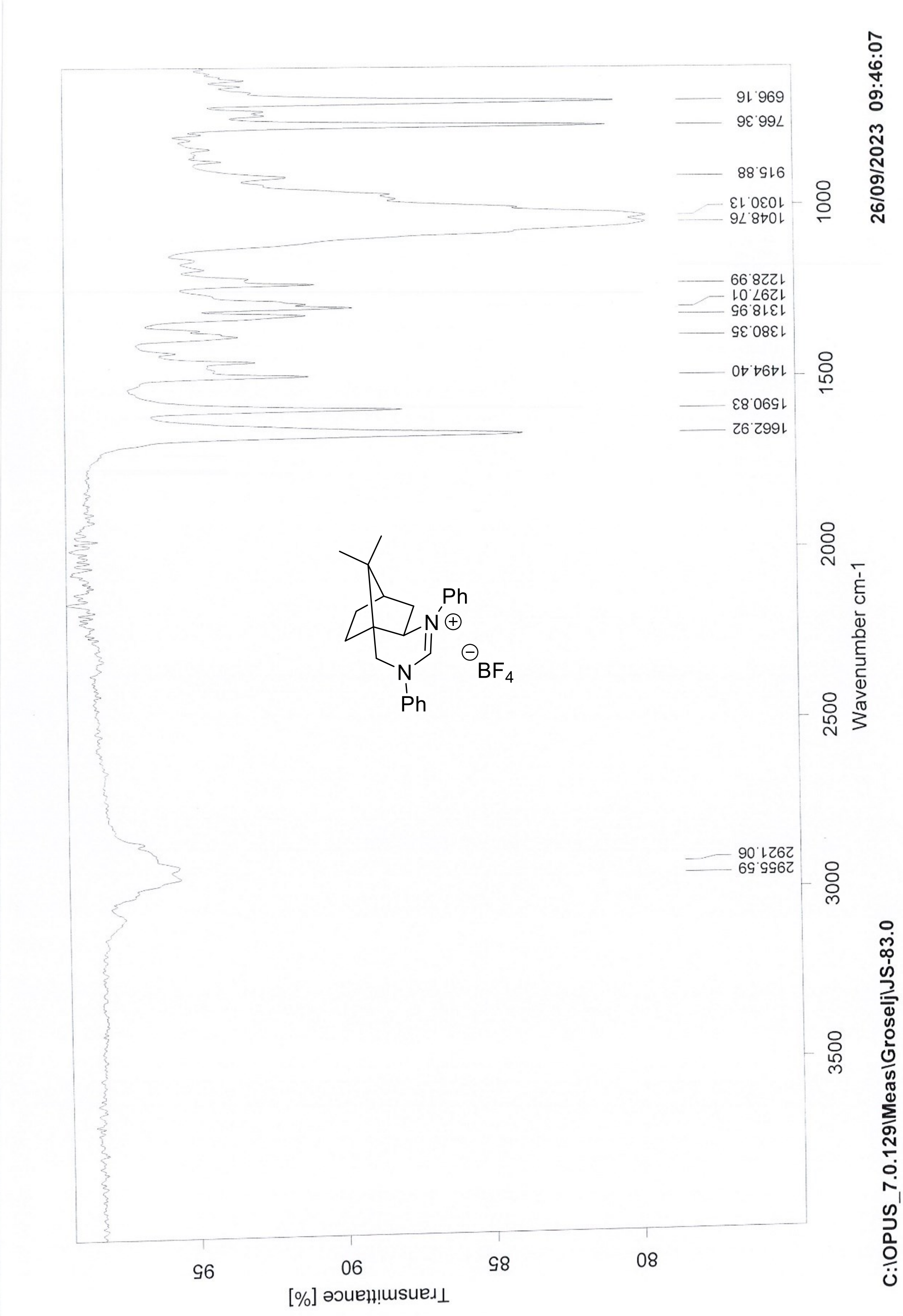

(4a*S*,7*R*)-9,9-Dimethyl-1,3-diphenyl-3,5,6,7,8,8a-hexahydro-4*H*-4a,7-methanoquinazolin-1-ium tetrafluoroborate (7c)

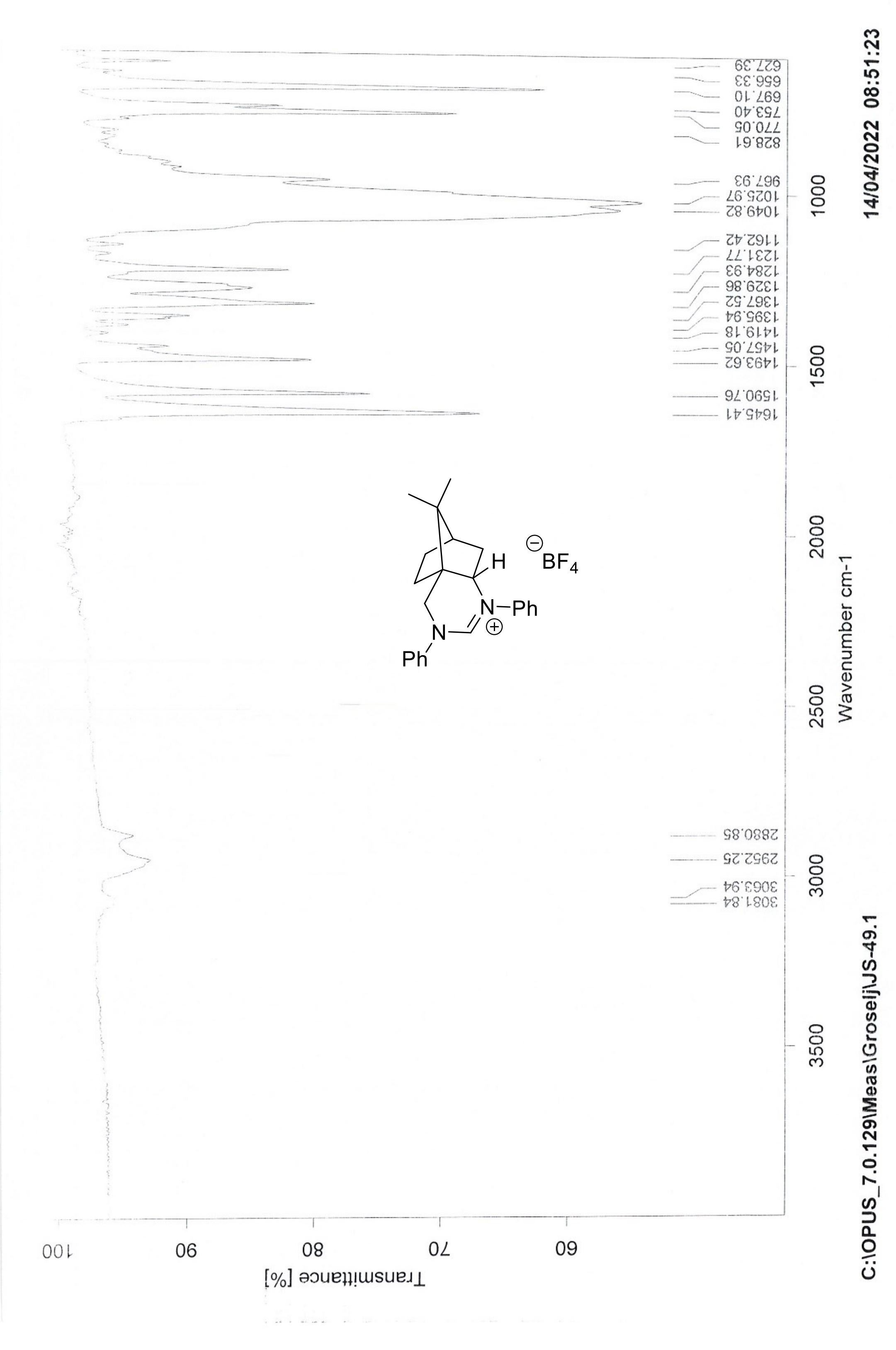

***N*-(((1*R*,2*R*,4*S*)-7,7-Dimethyl-2-(phenylamino)bicyclo[2.2.1]heptan-1-yl)methyl)-*N*-phenylformamide (8)**

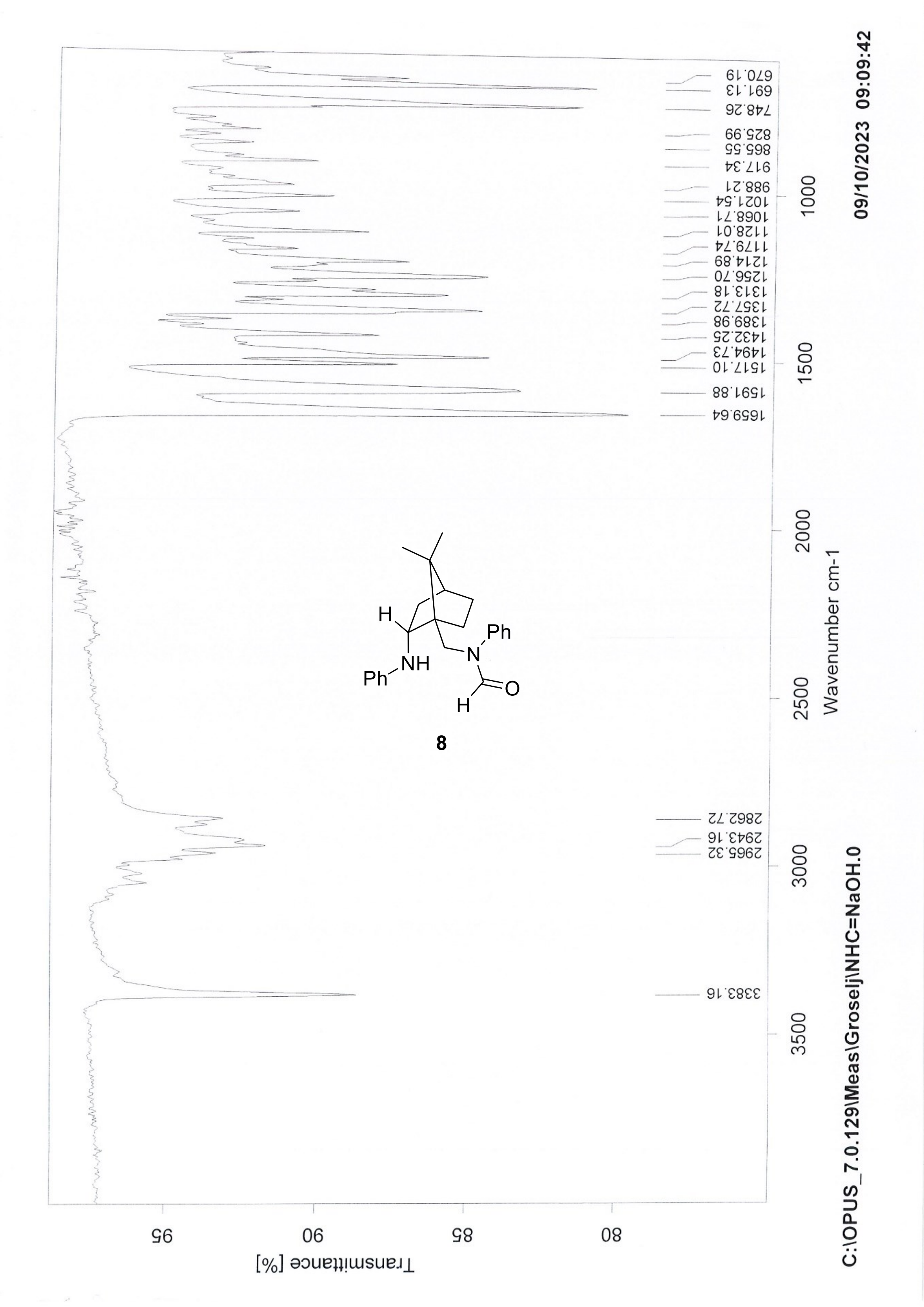

3. MS spectra

(1*R*,4*R*,*E*)-7,7-Dimethyl-*N*-phenyl-2-(phenylimino)bicyclo[2.2.1]heptane-1-carboxamide (4)

Qualitative Compound Report

**Data File**	JS-74_001.d	**Sample Name**	JS-74
**Sample Type**	Sample	**Position**	Vial 58
**Instrument Name**	US10310002	**User Name**	TOF-PC\admin
**Acq Method**	Bypass.m	**Acquired Time**	5/5/2022 1:15:33 PM
**IRM Calibration Status**	Success	**DA Method**	Damijana.m
**Comment**			

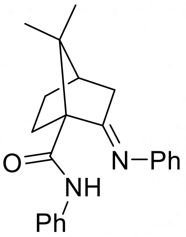

Compound Label	m/z	RT	Algorithm	Mass
Cpd 3: C22 H24 N2 O	333.1956	0.262	Find by Molecular Feature	332.1883

Compound Identification Results

Ion Mass	Calc Ion Mass	Difference	IonFormula	IonSpecies	Best
333.1956	333.1961	0.5	C22 H25 N2 O	(M+H)+	✓
333.1956	333.1948	-0.8	C20 H23 N5	(M+H)+	
333.1956	333.1966	1	C8 H27 N7 O7	(M+H)+	

MS Spectrum

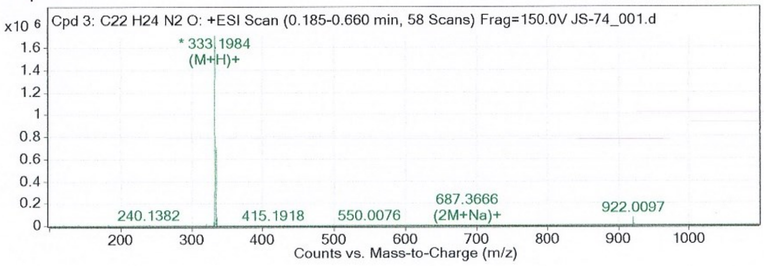

MFE MS Zoomed Spectrum

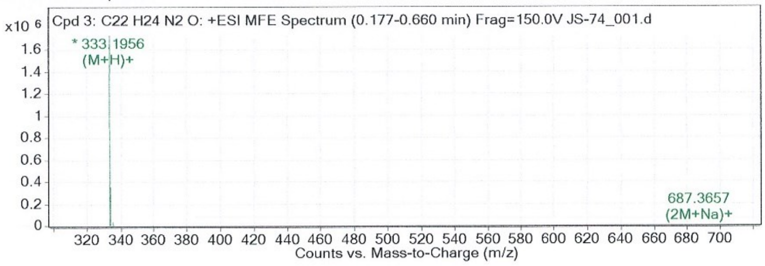

--- End Of Report ---

Agilent Technologies Page 1 of 1 Printed at: 1:18 PM on:5/5/2022

(1*R*,2*R*,4*R*)-7,7-Dimethyl-*N*-phenyl-2-(phenylamino)bicyclo[2.2.1]heptane-1-carboxamide (5a)

Qualitative Compound Report

**Data File**	JS-75_001.d	**Sample Name**	JS-75
**Sample Type**	Sample	**Position**	Vial 56
**Instrument Name**	US10310002	**User Name**	TOF-PC\admin
**Acq Method**	Bypass.m	**Acquired Time**	5/5/2022 1:10:53 PM
**IRM Calibration Status**	Success	**DA Method**	Damijana.m
**Comment**			

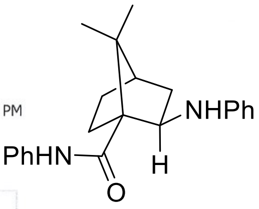

Compound Label	*m/z*	RT	Algorithm	Mass
Cpd 3: C22 H26 N2 O	335.2112	0.263	Find by Molecular Feature	334.204

Compound Identification Results

Ion Mass	Calc Ion Mass	Difference	IonFormula	IonSpecies	Best
335.2112	335.2118	0.6	C22 H27 N2 O	(M+H)+	✓
335.2112	335.2104	-0.7	C20 H25 N5	(M+H)+	

MS Spectrum

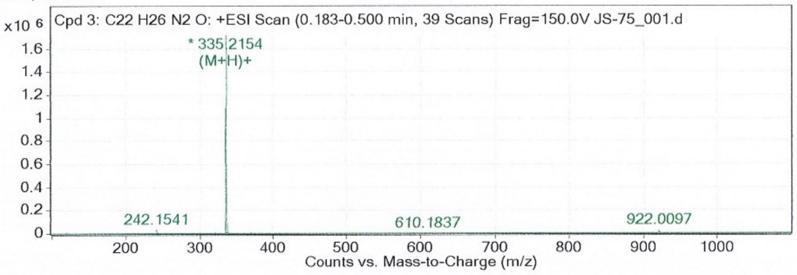

MFE MS Zoomed Spectrum

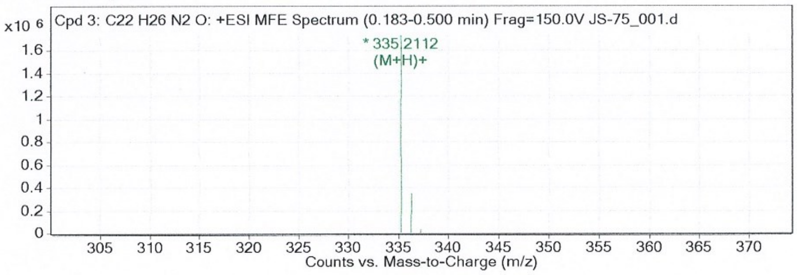

--- End Of Report ---

(1*S*,2*R*,4*R*)-7,7-Dimethyl-*N*-phenyl-1-((phenylamino)methyl)bicyclo[2.2.1]heptan-2-amine (6a)

Qualitative Compound Report

**Data File**	JS-18_001.d	**Sample Name**	JS-18
**Sample Type**	Sample	**Position**	Vial 54
**Instrument Name**	US10310002	**User Name**	TOF-PC\admin
**Acq Method**	Bypass.m	**Acquired Time**	5/5/2022 12:58:17 PM
**IRM Calibration Status**	Success	**DA Method**	Damijana.m
**Comment**			

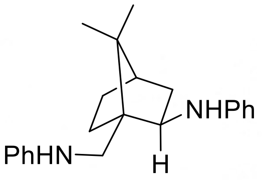

Compound Label	*m/z*	RT	Algorithm	Mass
Cpd 2: C22 H28 N2	321.2323	0.269	Find by Molecular Feature	320.225

Compound Identification Results

Ion Mass	Calc Ion Mass	Difference	IonFormula	IonSpecies	Best
321.2323	321.2325	0.2	C22 H29 N2	(M+H)+	✓

MS Spectrum

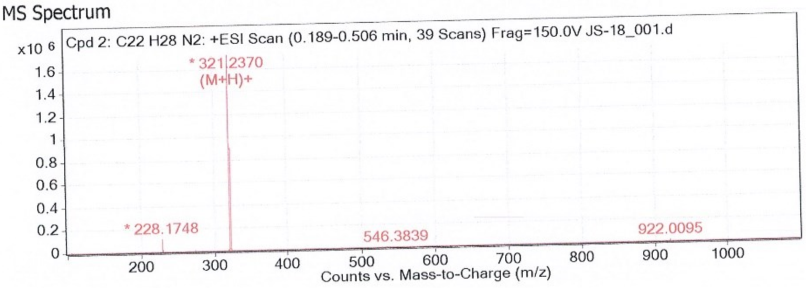

MFE MS Zoomed Spectrum

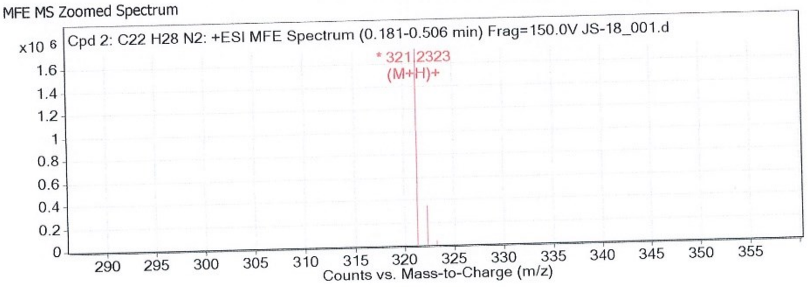

--- End Of Report ---

Agilent Technologies Page 1 of 1 Printed at: 1:08 PM on:5/5/2022

(1*S*,2*S*,4*R*)-7,7-Dimethyl-*N*-phenyl-1-((phenylamino)methyl)bicyclo[2.2.1] heptan-2-amine (6b)

Qualitative Compound Report

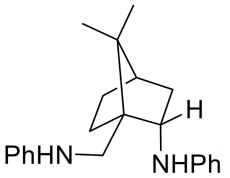

**Data File**	JS-90_002.d	**Sample Name**	JS-90
**Sample Type**	Sample	**Position**	Vial 65
**Instrument Name**	US10310002	**User Name**	TOF-PC\admin
**Acq Method**	Bypass.m	**Acquired Time**	5/5/2022 2:15:28 PM
**IRM Calibration Status**	Success	**DA Method**	Damijana.m
**Comment**			

Compound Label	*m/z*	RT	Algorithm	Mass
Cpd 2: C22 H28 N2	321.2323	0.263	Find by Molecular Feature	320.225

Compound Identification Results

Ion Mass	Calc Ion Mass	Difference	IonFormula	IonSpecies	Best
321.2323	321.2325	0.3	C22 H29 N2	(M+H)+	✓

MS Spectrum

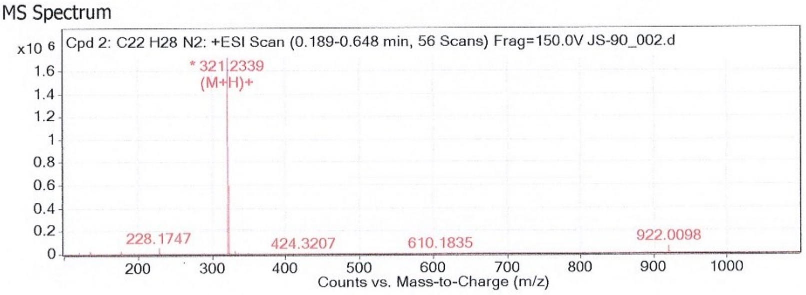

MFE MS Zoomed Spectrum

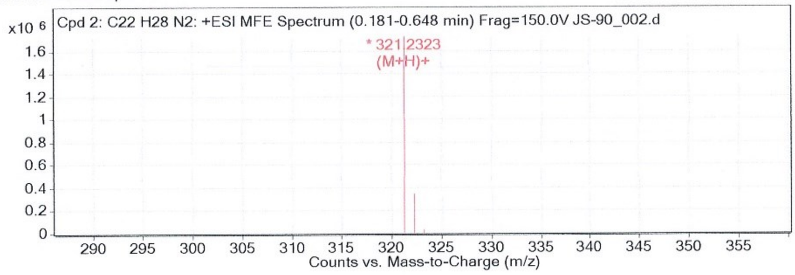

--- End Of Report ---

Agilent Technologies   Page 1 of 1   Printed at: 2:17 PM on:5/5/2022

(7*R*,8a*R*)-9,9-Dimethyl-1,3-diphenyl-3,5,6,7,8,8a-hexahydro-4*H*-4a,7-methanoquinazolin-1-ium chloride (7a)

Qualitative Compound Report

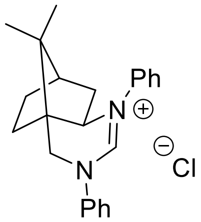

**Data File**	JS-10_001.d	**Sample Name**	JS-10
**Sample Type**	Sample	**Position**	Vial 12
**Instrument Name**	US10310002	**User Name**	TOF-PC\admin
**Acq Method**	Bypass.m	**Acquired Time**	12/16/2021 2:02:40 PM
**IRM Calibration Status**	Success	**DA Method**	Damijana.m
**Comment**			

Compound Label	*m/z*	RT	Algorithm	Mass
Cpd 4: C23 H26 N2	331.2166	0.263	Find by Molecular Feature	330.2093

Compound Identification Results

Ion Mass	Calc Ion Mass	Difference	IonFormula	IonSpecies	Best
331.2166	331.2169	0.3	C23 H27 N2	(M+H)+	✓
331.2166	331.2174	0.8	C9 H29 N7 O6	(M+H)+	

MS Spectrum

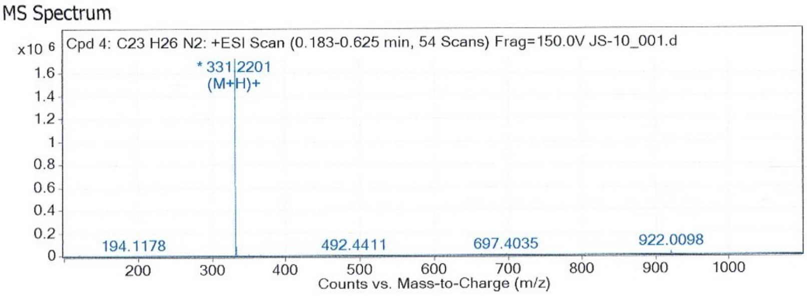

MFE MS Zoomed Spectrum

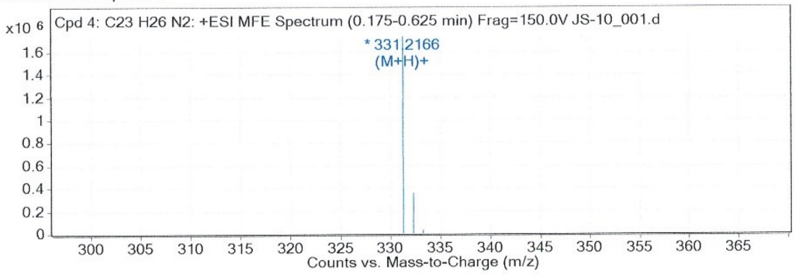

--- End Of Report ---

Agilent Technologies | Page 1 of 1 | Printed at: 2:03 PM on:12/16/2021

(7*R*,8a*R*)-9,9-Dimethyl-1,3-diphenyl-3,5,6,7,8,8a-hexahydro-4*H*-4a,7-methanoquinazolin-1-ium tetrafluoroborate (7b)

Qualitative Compound Report

**Data File**	JS-47_001.d	**Sample Name**	JS-47
**Sample Type**	Sample	**Position**	Vial 12
**Instrument Name**	US10310002	**User Name**	TOF-PC\admin
**Acq Method**	Bypass.m	**Acquired Time**	3/1/2022 9:54:04 AM
**IRM Calibration Status**	Success	**DA Method**	Damijana.m
**Comment**			

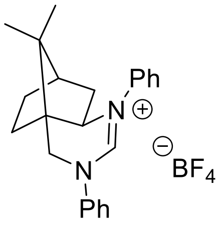

Compound Label	*m/z*	RT	Algorithm	Mass
Cpd 1: C23 H26 N2	331.217	0.257	Find by Molecular Feature	330.2098

Compound Identification Results

Ion Mass	Calc Ion Mass	Difference	IonFormula	IonSpecies	Best
331.217	331.2169	-0.2	C23 H27 N2	(M+H)+	✓
331.217	331.2167	-0.4	C18 H26 F N5	(M+H)+	
331.217	331.2174	0.4	C9 H29 N7 O6	(M+H)+	
331.217	331.2165	-0.5	C13 H25 F2 N8	(M+H)+	
331.217	331.2165	-0.5	C14 H31 F2 N O5	(M+H)+	
331.217	331.2176	0.6	C10 H26 F3 N8 O	(M+H)+	
331.217	331.2163	-0.7	C8 H24 F3 N11	(M+H)+	
331.217	331.2178	0.8	C15 H27 F2 N5 O	(M+H)+	
331.217	331.218	1	C20 H28 F N2 O	(M+H)+	
331.217	331.2187	1.7	C10 H25 N11 O2	(M+H)+	

MS Spectrum

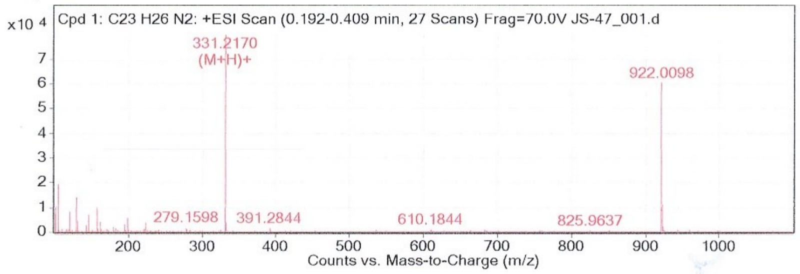

MFE MS Zoomed Spectrum

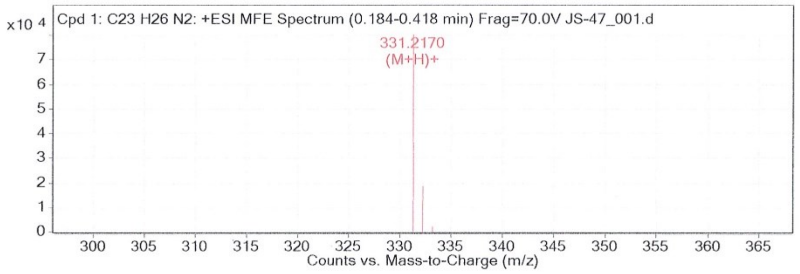

--- End Of Report ---

Agilent Technologies Page 1 of 1 Printed at: 10:00 AM on:3/1/2022

(4a*S*,7*R*)-9,9-Dimethyl-1,3-diphenyl-3,5,6,7,8,8a-hexahydro-4*H*-4a,7-methanoquinazolin-1-ium tetrafluoroborate (7c)

Qualitative Compound Report

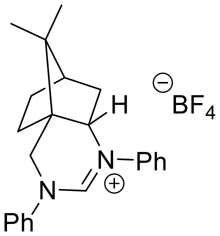

**Data File**	JS-49_001.d	**Sample Name**	JS-49
**Sample Type**	Sample	**Position**	Vial 55
**Instrument Name**	US10310002	**User Name**	TOF-PC\admin
**Acq Method**	Bypass.m	**Acquired Time**	5/5/2022 1:08:31 PM
**IRM Calibration Status**	Success	**DA Method**	Damijana.m
**Comment**			

Compound Label	*m/z*	RT	Algorithm	Mass
Cpd 3: C23 H26 N2	331.2163	0.267	Find by Molecular Feature	330.2091

Compound Identification Results

Ion Mass	Calc Ion Mass	Difference	IonFormula	IonSpecies	Best
331.2163	331.2169	0.6	C23 H27 N2	(M+H)+	✓
331.2163	331.2174	1.1	C9 H29 N7 O6	(M+H)+	

MS Spectrum

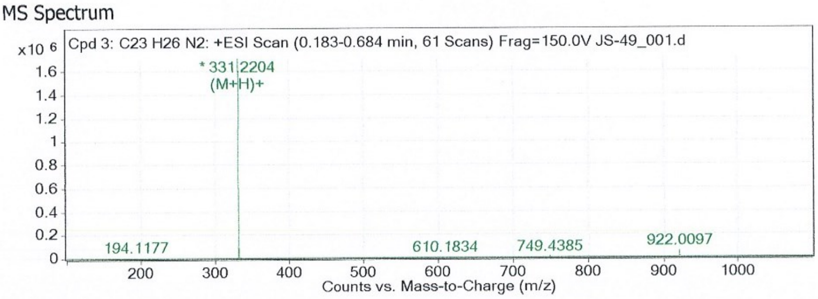

MFE MS Zoomed Spectrum

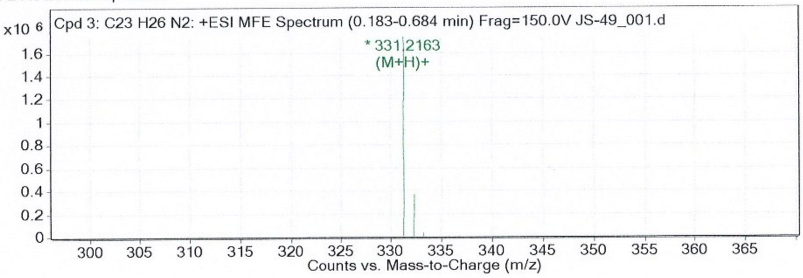

--- End Of Report ---

Agilent Technologies Page 1 of 1 Printed at: 1:11 PM on:5/5/2022

N-(((1R,2R,4S)-7,7-Dimethyl-2-(phenylamino)bicyclo[2.2.1]heptan-1-yl)methyl)-N-phenylformamide (8)

Qualitative Compound Report

**Data File**	NHC+NaOH_001.d	**Sample Name**	NHC+NaOH
**Sample Type**	Sample	**Position**	Vial 31
**Instrument Name**	US10310002	**User Name**	TOF-PC\admin
**Acq Method**	Bypass.m	**Acquired Time**	11/2/2023 10:46:38 AM
**IRM Calibration Status**	Success	**DA Method**	Damijana.m
**Comment**			

Compound Label	*m/z*	RT	Algorithm	Mass
Cpd 2: C23 H28 N2 O	349.2275	0.256	Find by Molecular Feature	348.2207

Compound Identification Results

Ion Mass	Calc Ion Mass	Difference	IonFormula	IonSpecies	Best
349.2275	349.2274	0	C23 H29 N2 O	(M+H)+	✓
349.2275	349.2261	-1.4	C21 H27 N5	(M+H)+	
349.2275	349.2293	1.8	C10 H27 N11 O3	(M+H)+	

MS Spectrum

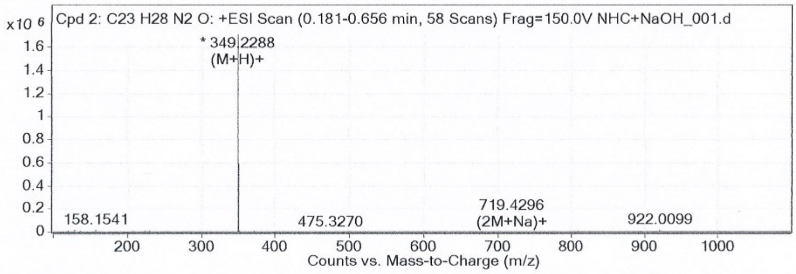

MFE MS Zoomed Spectrum

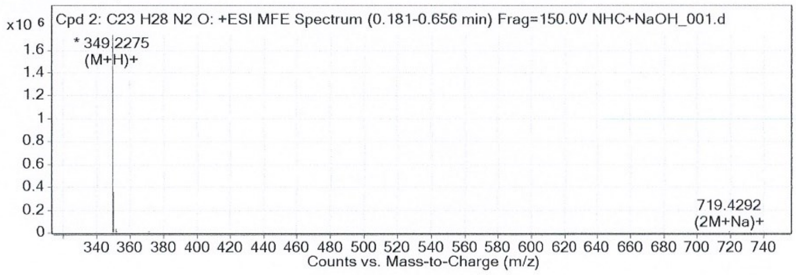

--- End Of Report ---

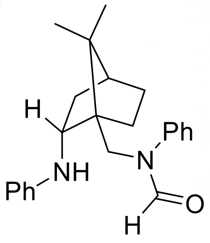

8

4. Structure Determination by NMR

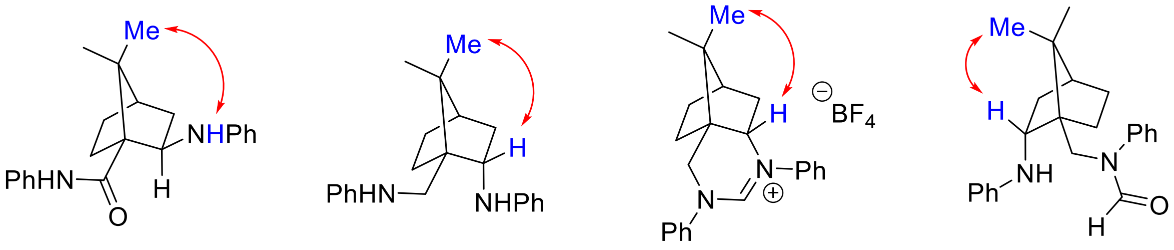

5a **6b** **7c** **8**

Figure S1. Determination of the absolute configuration at the C-2 based on the observed NOE correlation spectroscopy cross peaks.

NOESY spectra of compound **5a**

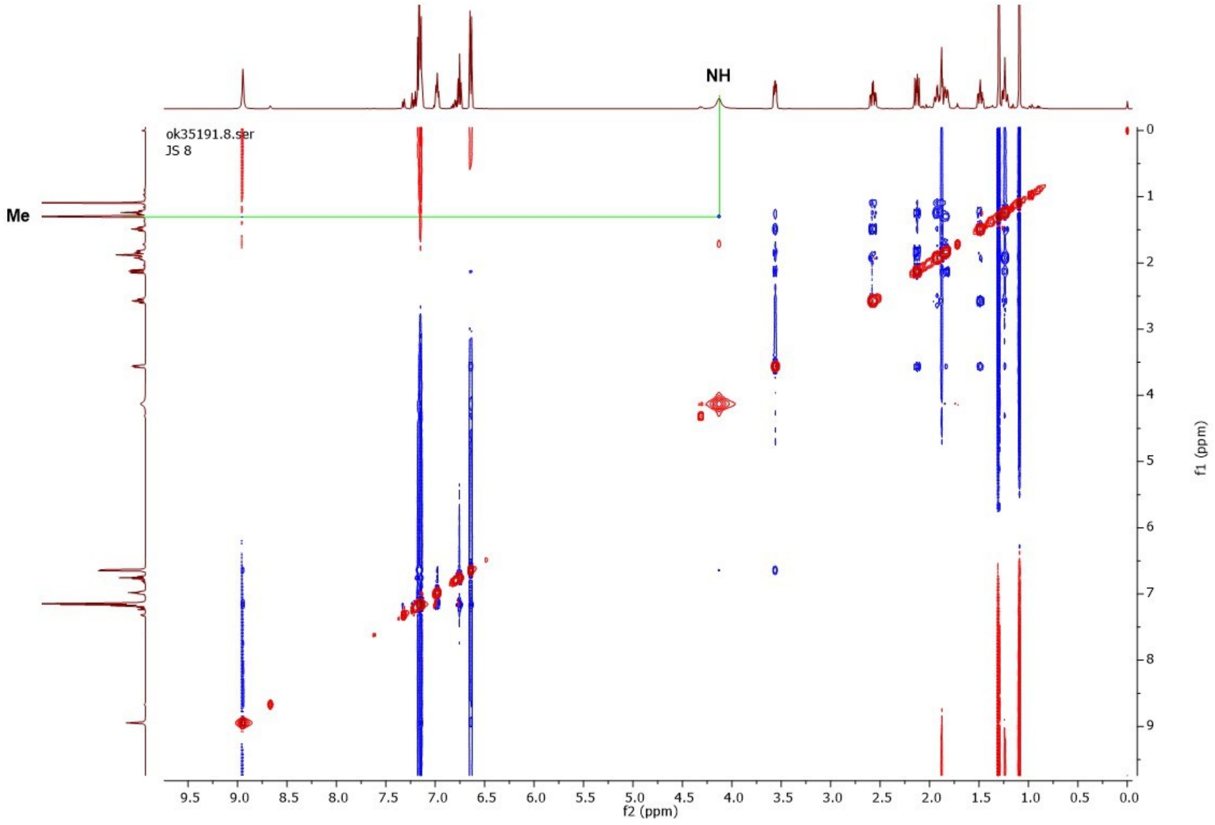

NOESY spectra of compound **6a**

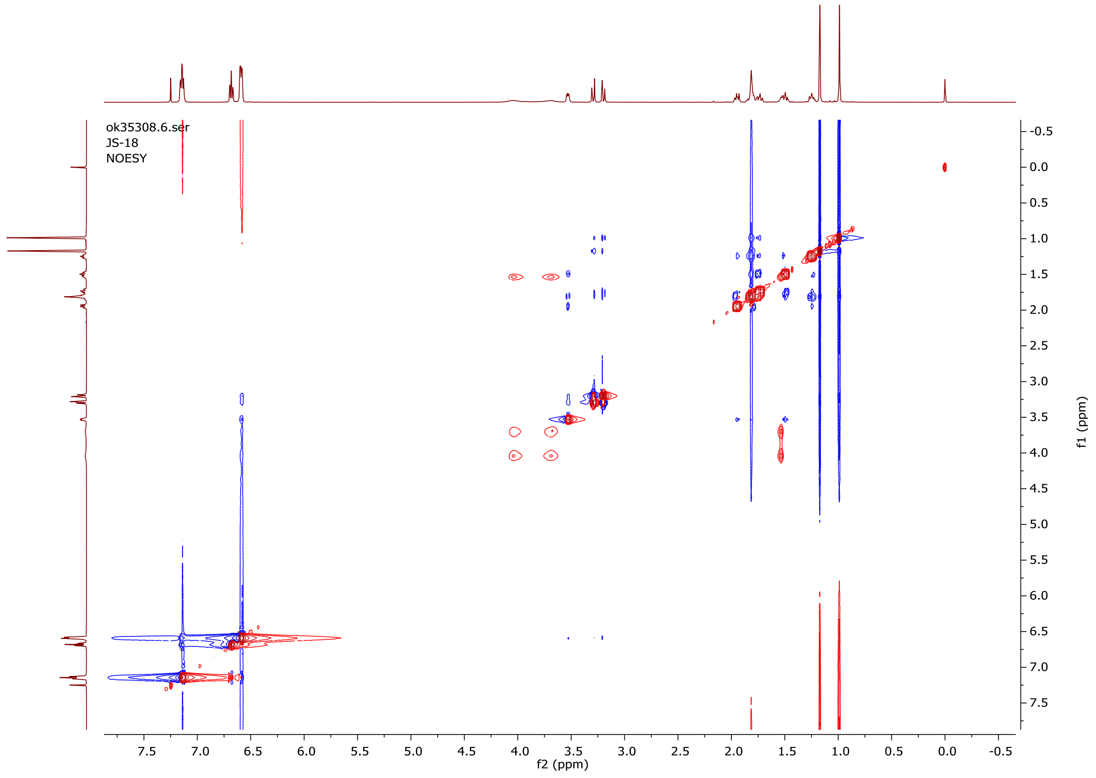

NOESY spectra of compound **6b**

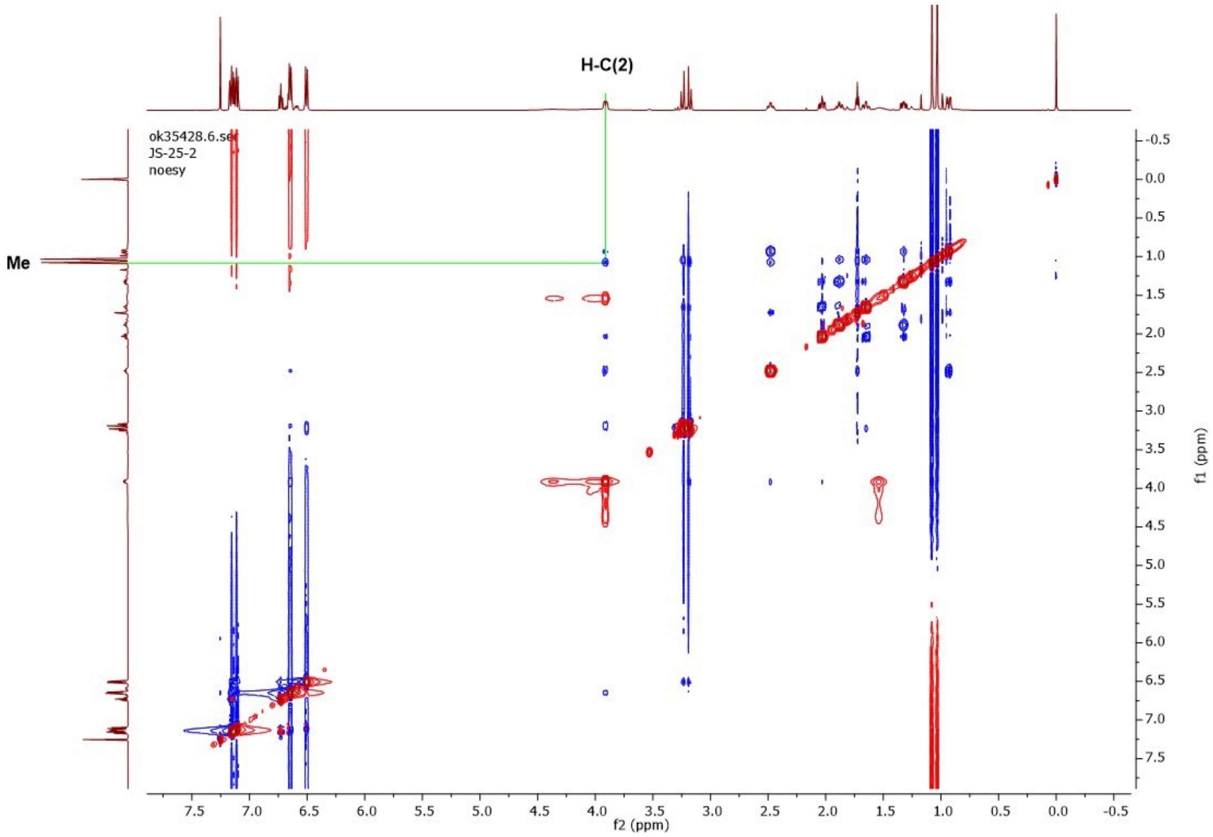

NOESY spectra of compound **7a**

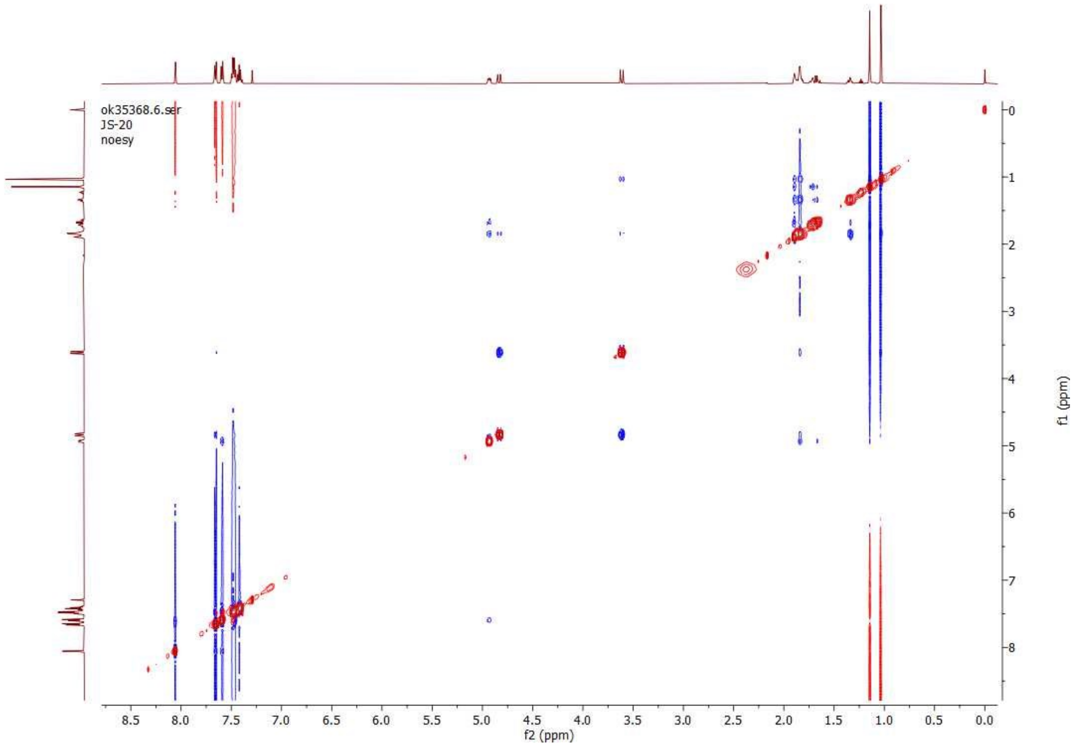

NOESY spectra of compound **7b**

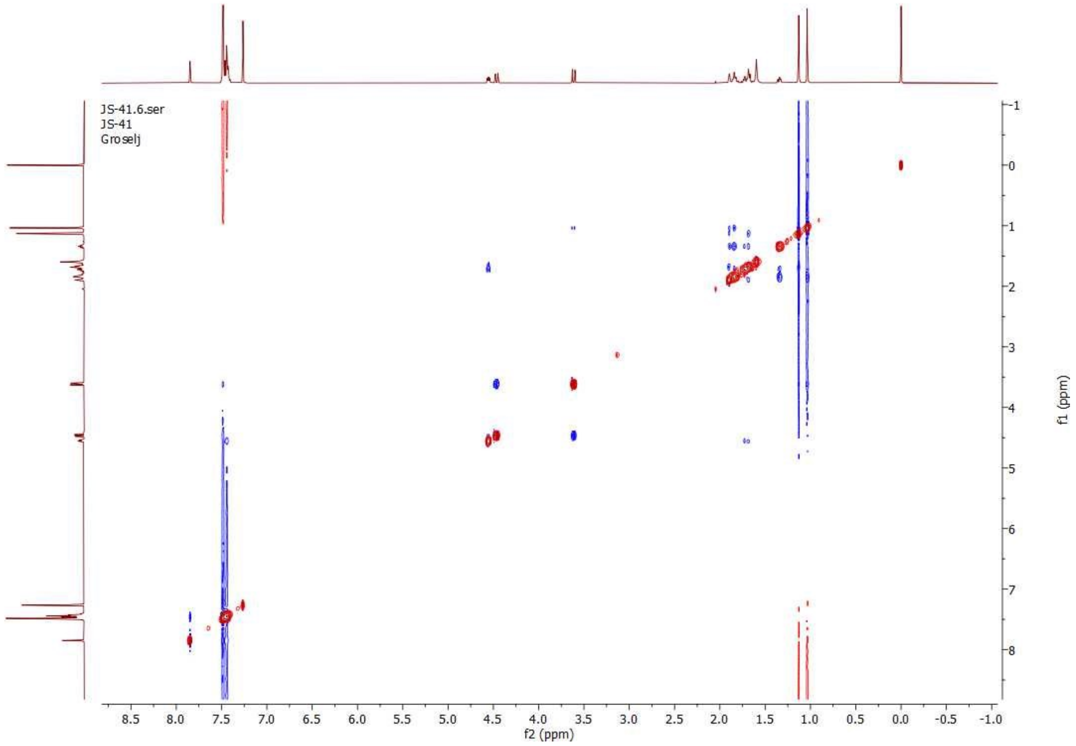

NOESY spectra of compound **7c**

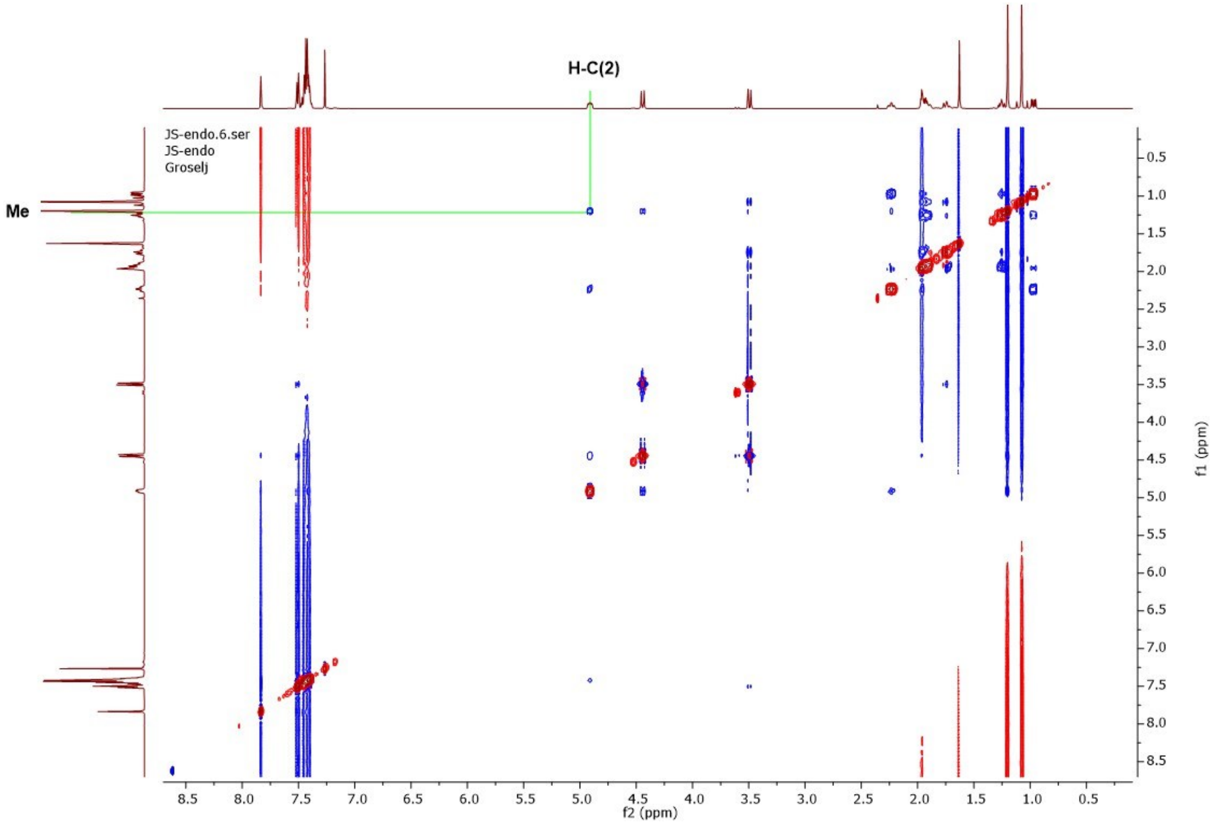

NOESY spectra of compound **8**

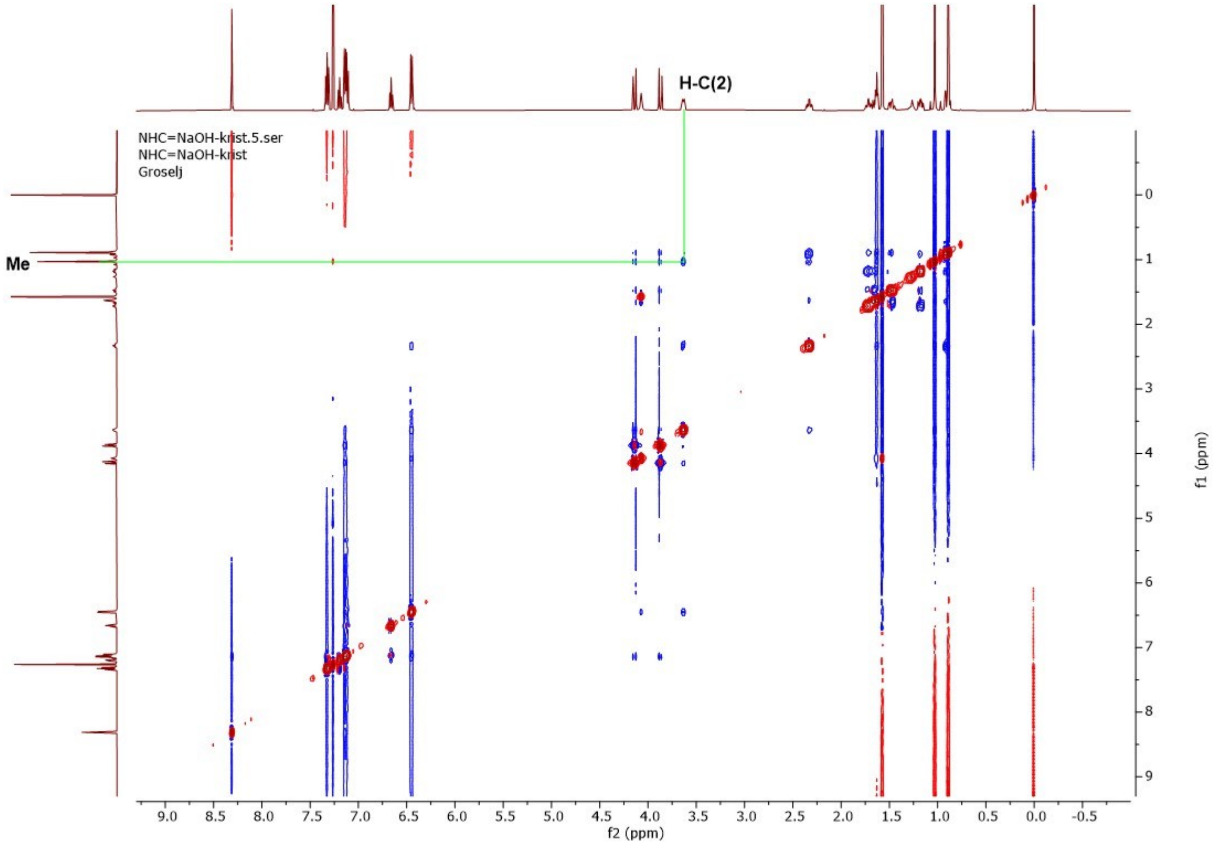

5. Structure determination by X-ray diffraction analysis

Table S1. Crystal data and structure refinement for compound **4**.

Empirical formula	$C_{44}H_{48}N_4O_2$
Formula weight	664.86
Temperature/K	149.4(8)
Crystal system	monoclinic
Space group	$P2_1$
a [Å^3]	11.86450(10)
b [Å^3]	10.25640(10)
c [Å^3]	15.9056(2)
α [°]	90
β [°]	109.3460(10)
γ [°]	90
V [Å^3]	1826.22(3)
Z	2
ρ_{calc} [g/cm^3]	1.209
μ [mm^{-1}]	0.579
F(000)	712.0
Crystal size/mm^3	0.8 × 0.5 × 0.4
Radiation	CuKα (λ = 1.54184)
Reflections collected	43372
Independent reflections	7278
R_{int}	0.0530
Data/restraints/parameters	7278/1/456
GOF	1.059
R_1, wR_2 [I>=2σ (I)]	0.0316, 0.0860
R_1, wR_2 (all data)	0.0321, 0.0867
$(\Delta\rho)_{max}$ [e Å^{-3}]	0.25
$(\Delta\rho)_{min}$ [e Å^{-3}]	–0.17
Flack parameter	0.08(9)

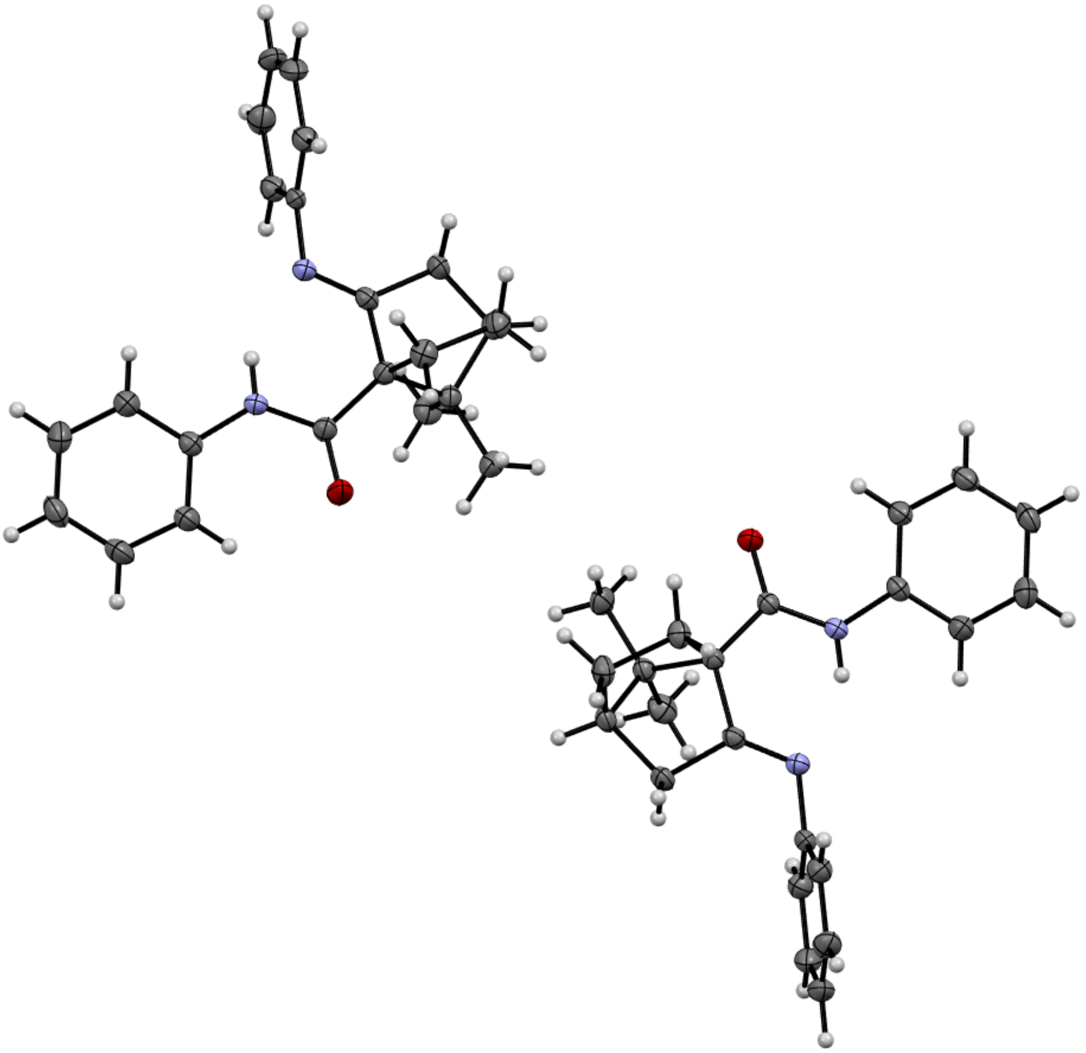

Figure S2. Molecular structure of compound **4**. Thermal ellipsoids are shown at 50% probability.

Table S2. Crystal data and structure refinement for compound **7a**.

Empirical formula	$C_{23}H_{27}ClN_2$
Formula weight	446.49
Temperature/K	150.00(10)
Crystal system	Hexagonal
Space group	$P6_3$
a [Å^3]	13.76552(13)
b [Å^3]	13.76552(13)
c [Å^3]	21.1278(2)
α [°]	90
β [°]	90
γ [°]	120
V [Å^3]	3467.12(7)
Z	6
ρ_{calc} [g/cm^3]	1.283
μ [mm^{-1}]	3.671
F(000)	1408.0
Crystal size/mm^3	0.3 × 0.2 × 0.2
Radiation	CuKα (λ = 1.54184)
Reflections collected	97691
Independent reflections	4793
R_{int}	0.0560
Data/restraints/parameters	4793/1/262
GOF	1.042
R_1, wR_2 [I>=2σ (I)]	0.0285, 0.0769
R_1, wR_2 (all data)	0.0294, 0.0780
$(\Delta\rho)_{max}$ [e Å^{-3}]	0.18
$(\Delta\rho)_{min}$ [e Å^{-3}]	–0.21
Flack parameter	–0.021(6)

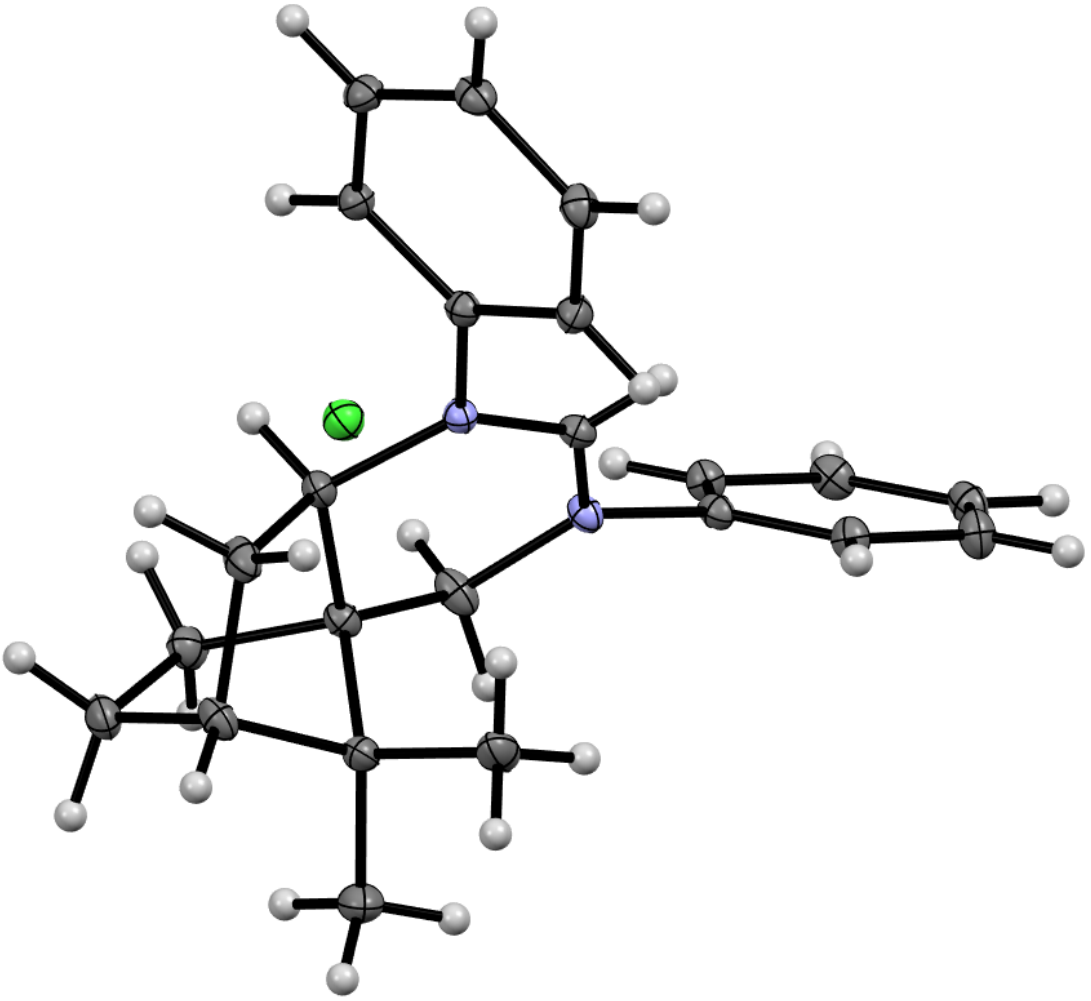

Figure S3. Molecular structure of compound **7a**. Thermal ellipsoids are shown at 50% probability.

Table S3. Crystal data and structure refinement for **8**.

Empirical formula	$C_{23}H_{28}N_2O$
Formula weight	348.47
Temperature/K	150.00(10)
Crystal system	Orthorombic
Space group	$P2_12_12_1$
a [Å^3]	7.7148(4)
b [Å^3]	12.8413(6)
c [Å^3]	18.9407(8)
α [°]	90
β [°]	90
γ [°]	90
V [Å^3]	1876.42(15)
Z	4
ρ_{calc} [g/cm^3]	1.234
μ [mm^{-1}]	0.075
F(000)	752.0
Crystal size/mm^3	0.4 × 0.3 × 0.2
Radiation	MoKα (λ = 0.71073)
Reflections collected	19268
Independent reflections	5035
R_{int}	0.0393
Data/restraints/parameters	5035/0/238
GOF	1.021
R_1, wR_2 [I>=2σ (I)]	0.0416, 0.0881
R_1, wR_2 (all data)	0.0549, 0.0954
$(\Delta\rho)_{max}$ [e Å^{-3}]	0.26
$(\Delta\rho)_{min}$ [e Å^{-3}]	–0.19
Flack parameter	0.2(6)

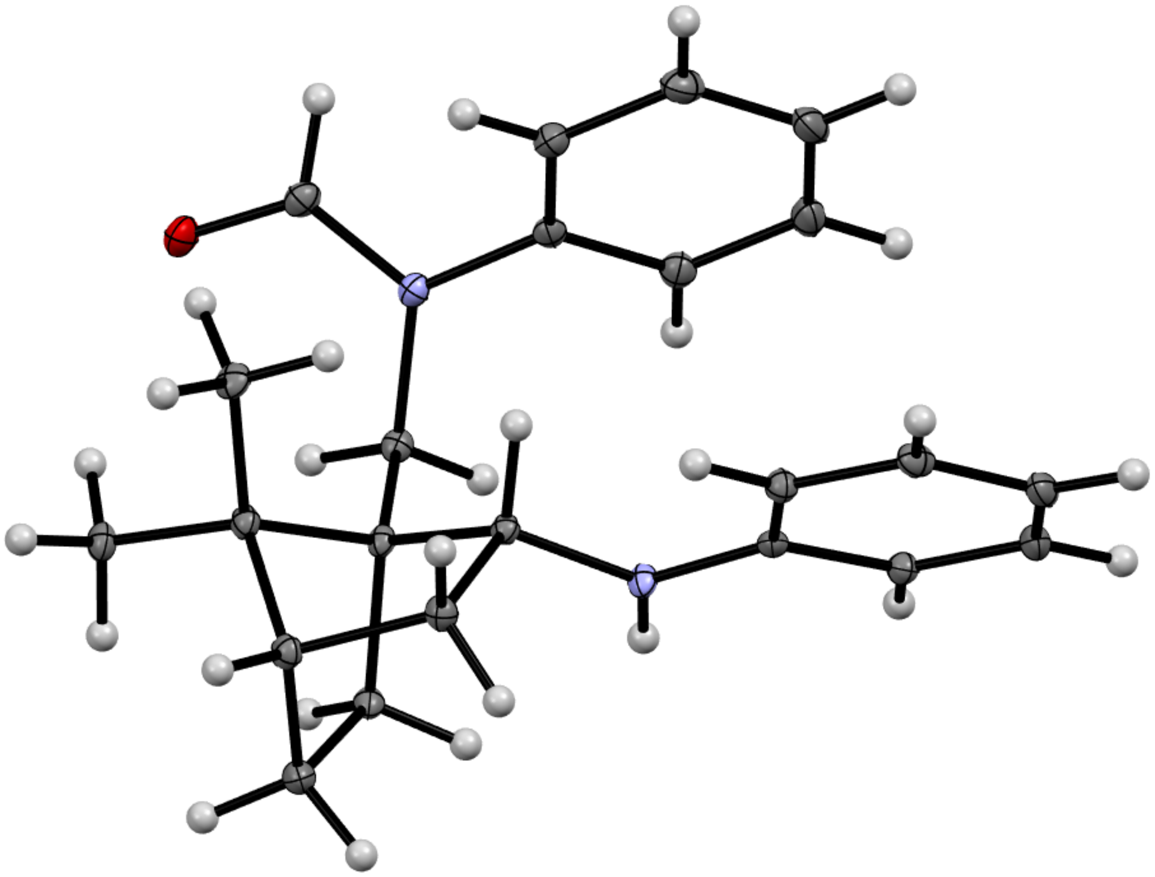

Figure S4. Molecular structure of compound **8**. Thermal ellipsoids are shown at 50% probability.
